# Emotion processing difficulties in ADHD: a Bayesian meta-analysis study

**DOI:** 10.1007/s00787-025-02647-3

**Published:** 2025-01-24

**Authors:** Ana-María Soler-Gutiérrez, Alberto J. Sánchez-Carmona, Jacobo Albert, José Antonio Hinojosa, Samuele Cortese, Alessio Bellato, Julia Mayas

**Affiliations:** 1 Faculty of Psychology, Universidad Nacional de Educación a Distancia (UNED), Despacho 2.36 bis, Calle Juan del Rosal, 10, 28040 Madrid, Spain; 2 Escuela Internacional de Doctorado de la UNED (EIDUNED), Universidad Nacional de Educación a Distancia (UNED), Madrid, Spain; 3 Centro Neuromottiva, 28016 Madrid, Spain; 4 Faculty of Psychology, Universidad Autónoma de Madrid, Madrid, Spain; 5 Instituto Pluridisciplinar, Universidad Complutense de Madrid, Madrid, Spain; 6 Faculty of Psychology, Universidad Complutense de Madrid, Madrid, Spain; 7 Centro de Investigación Nebrija en Cognición (CINC), Universidad Nebrija, Madrid, Spain; 8 School of Psychology, University of Southampton, Southampton, UK; 9 Centre for Innovation in Mental Health, University of Southampton, Southampton, UK; 10 Solent NHS Trust, Southampton, UK; 11 Clinical and Experimental Sciences (CNS and Psychiatry), Faculty of Medicine, University of Southampton, Southampton, UK; 12 Hassenfeld Children’s Hospital at NYU Langone, New York University Child Study Center, New York, NY USA; 13 DiMePRe-J-Department of Precision and Rigenerative Medicine-Jonic Area, Università degli Studi di Bari “Aldo Moro”, Bari, Italy; 14 Institute for Life Sciences, University of Southampton, Southampton, UK; 15 School of Psychology, University of Nottingham, Semenyih, Malaysia; 16 Mind and Neurodevelopment (MiND) Research Group, University of Nottingham, Semenyih, Malaysia

**Keywords:** ADHD, Emotional processing, Emotion recognition, Meta-analysis

## Abstract

We investigated whether there is an emotional processing deficit in ADHD and whether this only applies to specific emotional categories. In this PRISMA-compliant systematic review based on a pre-registered protocol (https://osf.io/egp7d), we searched MEDLINE, PsycINFO, ERIC, Scopus and Web of Science databases until 3rd December 2023, to identify empirical studies comparing emotional processing in individuals meeting DSM (version III to 5-TR) or ICD (version 9 or 10) criteria for Attention Deficit/Hyperactivity Disorder (ADHD) and in a non-psychiatric control group. Study quality was assessed with the Appraisal tool for Cross-Sectional Studies (AXIS). Eighty studies were included and meta-analysed (encompassing 6191 participants and 465 observations). Bayesian meta-analyses were conducted to compare individuals with ADHD and non-psychiatric controls on overall emotional processing measures (meta-analysis 1) and across emotional categories (meta-analysis 2). The type of stimulus employed, outcome measurement reported, age, sex, and medication status were analysed as moderators. We found poorer performance in both overall emotion processing (*g* =  − 0.65) and across emotional categories (anger *g* =  − 0.37; disgust *g* =  − 0.24; fear *g* =  − 0.37; sadness *g* =  − 0.34; surprise *g* =  − 0.26; happiness/positive *g* =  − 0.31; negative *g* =  − 0.20; neutral *g* =  − 0.25) for individuals with ADHD compared to non-psychiatric controls. Scales items and accuracy outcome being the most effective moderators in detecting such differences. No effects of age, sex, or medication status were found. Overall, these results show that impaired emotional processing is a relevant feature of ADHD and suggest that it should be systematically assessed in clinical practice.

**Supplementary Information:**

The online version contains supplementary material available at 10.1007/s00787-025-02647-3.

## Introduction

Attention-Deficit/Hyperactivity Disorder (ADHD) is a neurodevelopmental disorder characterized by developmentally inappropriate, persistent and impairing inattention and/or hyperactivity/impulsivity [1]. These symptoms may be associated with poor quality of life, and risk of premature mortality if not properly identified and treated [2, 3]. ADHD is one of the most prevalent childhood-onset disorders, affecting around 5% of children and adolescents [4] and impairing symptoms persist into adulthood in up to 70% of those diagnosed in childhood [5]. ADHD is a complex and heterogeneous disorder, both etiologically and phenotypically, and its causal mechanisms are not fully understood [6, 7]. Current evidence suggests that some individuals with ADHD may experience difficulties in inhibitory control [8], working memory [9], and emotional functioning [10, 11] (see [7] for a review). Difficulties in emotion regulation, processing, and recognition are likely to negatively impact social relationships and quality of life of people with ADHD.

The mechanisms underlying emotional dysfunction in ADHD are still unclear [12–14]. There is evidence of altered activation of the limbic system (including amygdala) and prefrontal systems (including the medial prefrontal cortex) underlying emotion processing [15, 16] in ADHD. Associations between difficulties in emotion regulation and altered autonomic functioning (especially, reduced parasympathetic vagal control) have also been reported, but these are not specific to ADHD as they can characterise people with other psychiatric or neurodevelopmental disorders [17, 18]. Some studies found that inattention is specifically associated with difficulties in emotion recognition [19, 20], particularly in relation to anger and sadness [21, 22]. However, other studies did not find evidence supporting these findings [23], or reported associations between other ADHD symptoms (e.g., impulsivity) and emotional functioning deficits [24]. Yet other studies found no correlation with ADHD symptoms [19]. Based on this body of evidence, the nature and extent of emotional functioning deficits in ADHD is unclear.

Emotional processing engages multiple neural networks to identify important stimuli and influence emotional states and behaviors. It consists of three main subprocesses: 1) identification, which recognizes emotional cues and assesses their significance; 2) reaction, which activates psychological and behavioral responses based on the stimulus' valence; and 3) modulation, which applies strategies to regulate emotional reactions to reach specific goals [25]. Most studies investigating emotional processing focus on the first step, understanding the processing as emotional detection and recognition. While some studies found poorer performance in emotion processing tasks in both children (e.g., [26–28]) and adults (e.g., [29, 30]) with ADHD, other studies failed to replicate these findings (e.g., [31–34]). Nevertheless, a meta-analysis of 77 studies (published up to 2015) in children and adolescents with ADHD found evidence of an emotional information processing deficit contributing to socio-emotional functioning difficulties independent of co-occurring conduct or cognitive problems [13]. Data on adults are more limited but findings are in the same direction (emotion processing deficit in ADHD), with only six studies (up to 2019) investigating emotion processing in adults with ADHD [35]. Difficulties in emotion recognition have also been detected in individuals with ADHD. Bora and Pantelis [36] meta-analysed 44 studies up to 2015 and found that people with ADHD, regardless of age and sex, showed difficulties in recognizing emotions during social cognition tasks or based on face- or voice-stimuli. This was corroborated by another meta-analysis of 21 studies (up to 2022) on vocal emotion recognition tasks [37], which found evidence of vocal emotion recognition deficits in ADHD, regardless of the emotion analysed.

A wide range of tools and outcome measures have been used to study emotion processing and recognition in ADHD, including various types of emotional stimuli that differ in terms of the type of emotion/valence they report on (e.g., discrete emotions or dimensional categories). Nevertheless, none of the previously discussed meta-analyses tried to disentangle the nature of emotion recognition/processing deficits in ADHD by investigating whether specific types of stimuli (e.g., faces, eyes, scenes, voices, or words) or outcome measures (e.g., performance accuracy, reaction time (RT), or other measures) modulate the differences found across studies between people with ADHD and controls. The present study therefore aimed to fill this gap by assessing whether there is an emotion processing deficit in ADHD and if such deficit is modulated by type of emotion assessed, as well as the type of stimulus used, and outcome measure collected. This is of relevance to better understand emotion functioning in ADHD, informing more personalised strategies to support the development of emotion recognition/processing skills tailored to specific subgroups of individuals with ADHD.

We used Bayesian meta-analysis, which allows to quantify the evidence in favour of both the null and the alternative hypothesis, and monitor evidence as data accumulate [38], therefore providing more robust results than traditional meta-analyses. The main objectives were: (a) investigating whether people with ADHD show alterations in overall emotion recognition/processing compared to neurotypical controls (meta-analysis 1, MA1), (b) exploring whether these differences are more evident for specific types of emotion assessed (e.g., happiness, sadness, fear, anger, disgust, surprise; positive, negative and neutral categories) (MA2), and (c) assessing whether variables such as sex, age, medication status, ADHD symptom severity, co-occurring conditions or diagnoses, type of outcome reported (accuracy, reaction time or other), or type of stimuli used (faces, voices, eyes, scenes, words, and scales) moderated the results (both for MA1 and MA2). Based on the reviewed literature, we expected to observe altered emotion processing and recognition in people with ADHD, compared to neurotypical controls, while we could not make any predictions regarding type/category of emotion investigated or other variables potentially moderating these effects.

## Methods

The reporting of this systematic review/meta-analysis followed the most updated PRISMA guidelines [39]. The protocol for this study was pre-registered on the OSF website, where the dataset is also available: https://osf.io/egp7d. The PRISMA checklist is included in Supplement 1.

### Search strategy and selection criteria

A systematic search was conducted on 3 December 2023 in MEDLINE, PsycINFO, ERIC, Scopus and Web of Science with the following pre-specified strategy, adapted for each database and limited to English language: (ADHD OR ADD OR “attention deficit hyperactivity disorder” OR “attention-deficit/hyperactivity disorder” OR “attention deficit disorder” OR “hyperkinetic disorder” OR “hyperkinetic syndrome”) AND (emotion* OR labil* OR affect* OR negative* OR irritability OR frustration OR “theory of mind” OR empathy). References from retrieved systematic reviews/meta-analyses were hand-searched to detect any relevant reference possibly missed with the electronic search. See Supplement 2 for a detailed search strategy description.

We included (a) original primary studies, (b) comparing people of any age meeting ADHD criteria according to DSM (II to 5-TR) or ICD (9,10) and a neurotypical non-psychiatric control group, and (c) reporting, either in the main text or supplementary materials, relevant information (e.g., means and standard deviations) of any available emotion recognition/processing measure derived from a task or a self-reported questionnaire/scale. Studies with unspecified ADHD diagnostic criteria, cohort studies without a control group, control groups including people with other psychiatric disorders, or emotion-induction experiments were excluded.

### Data extraction and outcomes

Records were screened based on title and abstract, first, and based on full text, then. Screening and data extraction was carried out by one author (AMSG). Queries were resolved by expert judgement (JM, JA, and JAH). We extracted relevant raw data (mean and standard deviations) including accuracy scores, reaction times, or other performance measures such as arousal-valence ratings and psychophysiological measurements, for the ADHD and control groups. As can be seen, it was possible to identify the presence of various effect sizes within each study. Thus, in order not to introduce any bias in the selection of any particular measure, all information was incorporated into the analysis. However, this measure raised the need to take into account the possible dependency between measures, integrating a new layer into the structure of the meta-analysis. Consequently, effect sizes were first nested within individual studies (level 2), and then aggregated together to form an overall effect size (level 3). AMSG used a Microsoft Excel spreadsheet for data extraction. Data from indirect measurements including emotion recognition/processing-relevant outcomes from cognitive tasks (e.g., n-back, Go/no-Go, Stroop, and continuous performance tasks), as well as self-report questionnaires/scales (e.g., Self-Assessment Manikin, and Toronto Alexithymia Scale), and direct measurements, such as tasks in which the type of emotion displayed must be explicitly recognised by the participant (e.g., Reading the Mind in the Eyes Test, RMET; Diagnostic Analysis of Nonverbal Behavior, DANVA; facial emotion recognition tasks), were extracted. Each outcome was classified by the type of emotional stimuli used (face, eyes, voice, scale/questionnaire), as well as by the type of emotion (happiness/positive, neutral, negative, sadness, angry, fear, disgust, and surprise). The categories “positive” and “negative” were used for studies where emotional categories (based on valence, e.g., positive or negative), but not a specific set of emotions, were used. We also extracted information about variables that might moderate the association between ADHD and emotion recognition/processing, such as age, sex, co-occurring conditions, medication status, and ADHD symptom severity. Study quality was assessed by AMSG using the Appraisal tool for Cross-Sectional Studies (AXIS; Supplement 3).

### Statistical analyses

All analyses were carried out using the *metafor* [40] (version 3.4–0), *brms* [41] (version 2.18.0) and *bayestestR* [42] (version 0.13.1) packages for the statistical software program R [43] (version 4.1.3). Hedge’s G (Standardised Mean Differences) were calculated (ADHD data vs control group data) to estimate differences between ADHD and non-ADHD groups on emotion processing outcomes; hence, negative effect sizes indicate poorer emotion processing in ADHD compared to the control group. Before fitting each model, an influence analysis (based on the criteria of Cook’s distance, hatvalues and dfbetas) was performed to detect possible outliers with respect to their role in the pooled effect size [44, 45].

Two Bayesian multilevel meta-analyses (MAs) were conducted to study differences in emotion recognition/processing between ADHD patients and non-psychiatric controls (MA1 was focused on overall measures of emotional recognition/processing, while MA2 focused on discrete emotions and valence dimensions). Effect sizes were first nested within individual studies, and then pooled together to form a global effect size. Publication bias was assessed by visually exploring the symmetry of the funnel plots and quantitatively by constructing a regression of the individual effect sizes on their corresponding standard errors [46]. Heterogeneity—associated with both the difference in true intra-cluster effect size and with inter-cluster variation, because of the multilevel nature of the analysis—was investigated via the I^2^ parameter [47]. Moderation analyses were also conducted, with the same Bayesian multilevel procedure used but including moderator variables as predictors in the models. Specifically, Age (mean), Sex (% males), Medication status (under medication, without medication/drug-naïve, washout period), Type of emotional stimuli (scales, scenes, faces, eyes, words, and voices) and Outcome measure used (accuracy, RTs, and other) were analysed as moderators for MA1. Type of stimuli (faces, eyes, voices, words, and scenes) and Outcome measurement (accuracy, RTs and other) were analysed as moderators for MA2.

Considering we adopted a Bayesian approach, a weakly informative prior was chosen given the lack of specific prior information, incorporating the possibility that certain values are more credible than others, but maintaining a general character that allows it to be applied to multiple contexts [48]. Concretely, the following parameters were chosen: $\mu \sim \aleph (\text{0,1})$; $\tau \sim HC(\text{0,0.5}$). In any case, to eliminate the presence of any bias and to test the robustness of the results obtained, a sensitivity analysis was performed. Thus, the results of the above analysis were compared with those associated with two different priors. Specifically, each model was evaluated twice more, but starting from a weak prior ($\mu \sim \aleph (\text{0,10})$ and from a vague prior ($\mu \sim \aleph (\text{0,100})$.

Bayesian models were interpreted in terms of different factors. Firstly, the confidence intervals that contained the true value of the parameter with a 95% probability (high density interval, HDI) were reported. In addition, we examined what percentage of the posterior distribution of the parameter was compatible with the hypothesis that it differed from zero (credibility). We also provided the evidence ratios associated with this hypothesis, which quantify the evidence provided by the estimate in favour of the effect versus the alternative interpretations. It was concluded that there was indeed a difference between groups if this HDI differed from the criterion (zero). However, this procedure would only allow the rejection of the null parameter, but not its acceptance. Therefore, to complete the decision making on effects, the procedure based on the region of practical equivalence (ROPE) was used [49]. This procedure consists of setting a range of values around the null value, which, in practical terms, would reflect the absence of effects. In our case, the ROPE was set between − 0.1 and + 0.1 around a zero value, on the scale of the standardised mean difference. Thus, the zero value was rejected if the 95% HDI does not overlap at all with the ROPE region. Conversely, if the 95% HDI fell within the ROPE region, the zero value was accepted. In any other case, the decision would be undecided. Beyond the criteria used to reject the null hypothesis, each analysis was accompanied by an assessment of the level of precision achieved. Concretely, the width of each confidence interval was compared with a practical threshold set at 80% of the ROPE region (0.16) [50, 51]. This precision assessment allowed for a proper weighting of the relevance of each conclusion, especially for the moderation analyses where smaller samples of studies were used. For moderation analyses, decision making regarding the null hypothesis (no differences with respect to the intercept or other levels of the moderator variable) was based on the assessment of the degree of overlap between the HDI and ROPE regions. Importantly, the scale of the continuous variables was adjusted to the standardised mean different scale, because of its impact on decision making based on ROPE region.

## Results

**Fig. 1:**
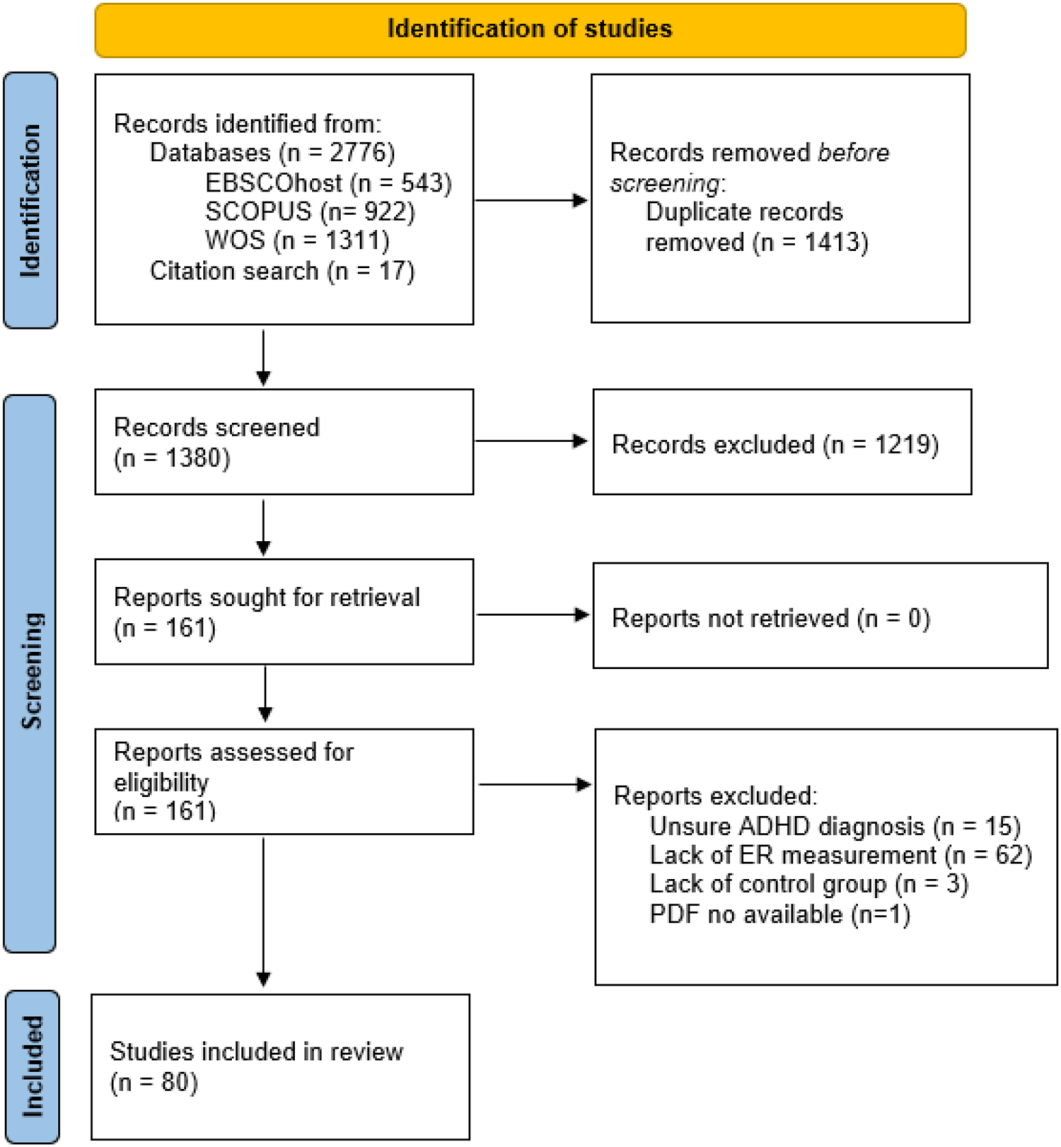
PRISMA flowchart. *Notes.* ER: Emotion Recognition/Processing

Of 1380 references initially screened, 161 full texts were assessed for eligibility (Fig. 1). A total of 80 studies (6191 participants in total, 53% with ADHD, 77% children/adolescents) met the inclusion criteria, from which 465 observations (effect sizes) were obtained. Table 1 provides detailed data about the studies included and Table 2 summarises the main characteristics of the studies. Sample sizes ranged from 20 to 364 participants, with the majority focusing on children and adolescents, and some covering age ranges as wide as 6 to 18 years. Only 53% of the studies specified the ADHD subtype/presentation, with the combined subtype/presentation being the most prevalent (65%). Furthermore, an under-representation of women was also observed (72% male participants) in line with the sex ratio seen in clinical practice, possibly accounted for, at least in part, by referral bias. In 74% of the studies, the presence or absence of comorbidity was reported. Additionally, behavioural problems, including conduct disorder (CD) and oppositional defiant disorder (ODD), were reported in 28.75% of the studies. In terms of ADHD medication status, 59% of studies indicated that a 24–48-h washout period required before the testing session, while 23% did not provide information about participants taking medication.

**Table 1:** Characteristics of studies included in the systematic review/meta-analysis

1st author and year	Sample size	Developmental stage	Emotion recognition/processing tool	Main findings reported
Albayrak 2022 [52]	41 ADHD; 43 controls	Children and adolescents	RMET	Scores: ADHD < Controls
Alperin 2017 [53]	49 ADHD; 60 controls	Adolescents	Emotional Faces Go/no-Go Task	Accuracy: ADHD < Controls; fear < neutral = happy RT: no effect group
Andrade 2012 [54]	39 ADHD; 25 controls	Children	Social information processing vignettes	Control group detected a significantly larger proportion of positive, negative, and neutral cues, after adjusting for conduct problems
Ayaz 2013 [55]	64 ADHD; 69 controls	Adolescents	RMET	Correct responses: ADHD < Controls
Balogh 2017 [56]	26 ADHD; 14 controls	Adults	Emotional Go/no-Go Task	Commission errors: ADHD > Controls for neutral and negative stimuli. No differences for positive valence. RT: no group differences
Basile 2018 [31]	39 ADHD; 42 controls	Children	Emotion Recognition Task	Accuracy: no group differences. ER confidence: ADHD > Controls. ER gamma resolution index (discriminating correct from incorrect responses): ADHD < Controls
Berenguer 2018 [57]	35 ADHD; 37 controls	Children	Emotion recognition (NEPSY-II)	ER scores: ADHD < Controls
Berggren 2016 [19]	32 ADHD; 32 controls	Children and adolescents	Facial affect recognition (FAR)	Overall FAR face accuracy: no group differences. Overall FAR eyes accuracy: ADHD < Controls. Overall RT: no group differences
Blaskey 2008 [58]	71 ADHD; 45 controls	Children	Chimeric Faces Test	In happy-neutral condition (for left-handers), control children showed the usual left-visual hemispace (LVH) bias, but ADHD combined subtype did not. Right-handers (controls and ADHD) showed the usual LVH bias in all conditions
Boakes 2008 [59]	24 ADHD; 24 controls	Children	Facial Affect Interpretation	Scores: ADHD < Controls interpretating disgust and fear No group differences regarding happiness, anger, and sadness
Bolat 2017 [60]	69 ADHD; 69 controls	Children and adolescents	Comprehension Test (CT)	CT scores: ADHD < Controls. ADHD-I = ADHD-C < Controls No ADHD subtype groups differences
Brotman 2010 [61]	18 ADHD; 37 controls	Children and adolescents	Facial emotion recognition	No differences rating subjective fear
Cadesky 2000 [62]	86 ADHD; 27 controls	Children	DANVA	Accuracy: ADHD < Controls on all emotions except anger. Analysis of the pattern of errors showed that ADHD made more errors but in a random manner, like the control group
Chronaki 2014 [63]	25 ADHD; 25 controls	Children	Vocal emotion recognition task	Accuracy: ADHD < Controls for angry voices ADHD tended to miscategorise angry voices as neutral
Conzelmann 2009 [64]	197 ADHD; 128 controls	Adults	IAPS rating	No differences between the control group and ADHD in time viewing or in valence and arousal ratings. ADHD showed a reduced emotional responding to pleasant stimuli
Corbett 2000 [65]	37 ADHD; 37 controls	Children	POFA & Prosody test	Accuracy: ADHD < Controls in both ER test. 85% of the variance was explained by POFA
Cortez-Carbonell 2017 [66]	30 ADHD; 30 controls	Adults	Facial emotion recognition	Accuracy: ADHD < Controls for the three facial expressions used (happiness, anger and neutral). RT: ADHD > Controls for anger, but not for happiness or neutral
Da Fonseca 2009 [26]	27 ADHD; 27 controls	Children and adolescents	Emotion recognition tasks	ER accuracy Experiment 1: ADHD < Controls. ER accuracy Experiment 2: ADHD < Controls. Object recognition Experiment 2: no group differences
Dan 2018 [67]	15 ADHD; 16 controls	Adults	Facial emotional expression morph task	ER threshold at baseline: no group differences. After sleep deprivation ADHD experienced an increased threshold for emotion recognition, while controls did not
Dan 2015 [68]	45 ADHD; 46 controls	Adolescents	Facial emotion recognition	Ratings: ADHD (combined) < Control (happy and neutral). RT: no group differences. Variability of RT and ratings: ADHD > Controls
Demirci 2016 [69]	60 ADHD; 60 controls	Children and adolescents	RMET	RMET accuracy: ADHD < Controls. ADHD-HI < ADHD-I = ADHD-C. Benton Face Recognition Test: ADHD < Controls
Demurie 2011 [70]	13 ADHD; 18 controls	Adolescents	RMET	Score: no group differences
Dini 2020 [71]	24 ADHD; 25 controls	Children	Facial emotion recognition	Accuracy and RT: no group differences Variance of RT: ADHD > Controls
Downs 2004 [72]	16 ADHD; 10 controls	Children	Emotional Understanding	Total correct: ADHD < Controls
Dyck 2001 [73]	35 ADHD; 36 controls	Children and adolescents	Facial cues test & Comprehensive Test	Empathic ability index (Facial cues & Comprehensive tests included): ADHD < Controls
Friedman 2003 [74]	31 ADHD; 32 controls	Adults	Emotional sensitivity subscale (SSI), Social context films & TAS-20	Emotional Sensitivity: no group differences. TAS-20: ADHD > Controls Social Context: ADHD < Controls in using affect-related words (unrelated to vocabulary skills or number of words to describe scenes). No group differences in Benton Test
Gonzalez-Gadea 2013 [32]	22 ADHD; 21 controls	Adults	RMET	RMET: no group differences
Grabemann 2013 [75]	20 ADHD; 20 controls	Adults	Florida Affect Battery	Correct responses: ADHD < Controls naming affects (incongruent condition). No differences in congruent condition
Greco 2021 [76]	20 ADHD; 21 controls	Children	Morphing Task—Human Faces	Latency: ADHD > Controls for happiness, anger, and disgust
Greenbaum 2009 [33]	30 ADHD; 34 controls	Children	MNTAP	No group difference in any subtest
Helfer 2021 [77]	43 ADHD; 46 controls	Adults	Facial emotion recognition	Accuracy: no group differences RT: ADHD > Controls (except for surprise)
Herrmann 2009 [78]	32 ADHD; 32 controls	Adults	View pictures IAPS while EEG	EPN amplitudes: ADHD < controls for positive stimuli condition No group differences for negative stimuli condition
Ibáñez 2014 [29]	16 ADHD; 41 controls	Adults	RMET	ADHD showed a trend toward reduced ER abilities compared to controls
Ibáñez 2011 [79]	10 ADHD; 10 controls	Adults	RMET	ADHD showed a trend toward reduced ER abilities compared to controls
Imanipour 2021 [80]	25 ADHD; 25 controls	Children	RMET	Correct responses: ADHD < Controls. In ADHD group, RMET was associated with biological motion discrimination
Kılınçel 2021 [81]	42 ADHD; 41 controls	Adolescents	Child Eyes Test	Scores: ADHD < Control
Kis 2017 [30]	28 ADHD; 29 controls	Adults	Tübinger Affect Battery (TAB)	TAB naming & discrimination: ADHD < Controls, particularly angry statements. TAB conflicting & matching: no group differences
Krauel 2009 [82]	18 ADHD; 15 controls	Children and adolescents	Perceptual and semantic task	No group differences in any perceptual or semantic task with neutral or emotional stimuli. RT variability: ADHD > Controls
Lee 2009 [83]	42 ADHD; 45 controls	Children	Facial emotion recognition	Accuracy: no group differences
Levy 2022 [84]	236 ADHD; 128 controls	Children and adolescents	RMET	Correct responses: no group differences In ADHD, high irritability predicted lower RMET accuracy
López-Martín 2013 [85]	20 ADHD; 20 controls	Children	Emo-distractors	Error rates: no group differences
López-Martín 2015 [86]	24 ADHD; 24 controls	Children	Emo-distractors (Go/no-Go)	No effect group in any measure
Maire 2018 [21]	40 ADHD; 40 controls	Children	Facial emotion recognition	ER scores: ADHD < Controls, only for full sadness. No group differences in geometric recognition. Inattention predicted lower anger recognition score
Manassis 2000 [87]	15 ADHD; 16 controls	Children	Emotional Word Test	No group differences for emotion words
Mauri 2020 [88]	20 ADHD; 25 controls	Children and adolescents	emo-CPT	RT: ADHD < Controls RT variability and false alarms: ADHD > Controls
Miller 2011 [89]	33 ADHD; 18 controls	Adults	DANVA	Fearful errors: ADHD-I > Controls. No differences between ADHD-I and ADHD-C, nor ADHD-C and controls. Inattention was associated with more errors
Miranda 2017 [27]	35 ADHD; 39 controls	Children	Affect Recognition (NEPSY-II)	Scores: ADHD < Controls. Affect recognition significantly correlated with Inhibit, Shift, Emotional control, and Behavioural Regulation Index of the BRIEF
Noordermeer 2020 [90]	82 ADHD; 82 controls	Adolescents	Facial and vocal emotion recognition	No group differences in any measurement
Özbaran 2018 [91]	100 ADHD; 100 controls	Children and adolescents	Faces Test & RMET	Face test and RMET scores: ADHD < Controls
Parke 2018 [92]	25 ADHD; 25 controls	Children	Affect Recognition (NEPSY-II)	Scores: ADHD < Controls
Passarotti 2010 [93]	14 ADHD; 19 controls	Children and adolescents	Facial emotion recognition	ADHD showed a nonsignificant trend (*p* = 0.06) for lower accuracy compared with controls. RT: no group differences
Passarotti 2010 [94]	15 ADHD; 14 controls	Children and adolescents	Emo-Stroop Task	Accuracy: no group differences RT: ADHD > Controls
Pelc 2006 [95]	30 ADHD; 30 controls	Children	Facial emotion recognition	Accuracy: ADHD < Controls for anger (70% intensity) and sadness (all intensities). ADHD showed significantly lower awareness of errors of anger and disgust compared with controls
Pitzianti 2017 [34]	23 ADHD; 20 controls	Children and adolescents	Emotion recognition (NEPSY-II)	No group differences
Plecevic 2021 [96]	31 ADHD; 29 controls	Children	GEES	Speech Emotional Expression and Attitude accuracy: ADHD < Controls for all emotions, except for joy
Rapport 2002 [97]	28 ADHD; 28 controls	Adults	Tachistoscope affect recognition & DANVA	Accuracy: ADHD < Controls for happy, angry, and fearful. RT: ADHD > Controls DANVA: ADHD < Controls for all measures
Saeedi 2014 [98]	30 ADHD; 30 controls	Children and adolescents	RMET	Score: ADHD < Controls
Sahin 2018 [28]	24 ADHD; 26 controls	Children	RMET	Score: ADHD < Controls
Schwenck 2013 [99]	56 ADHD; 28 controls	Children and adolescents	Morphing Task	Accuracy, RT, and RT variability: no group differences, included comparison between ADHD with and without medication
Semrud-Clikeman 2010 [100]	153 ADHD; 113 controls	Children and adolescents	CASP emotion cues	Scores: ADHD < Controls. ADHD symptoms predicted CASP emotional cues performing, but no CASP nonverbal cues
Serrano 2015 [101]	19 ADHD; 26 controls	Children	POFA & scene images	Face RT: Moderate to large effect sizes (ADHD > Controls). Face accuracy: moderate effect sizes for total and disgust (ADHD < Controls). Situations RT: moderate to large effect size, except for happy (ADHD > Controls). Situations accuracy: moderate for total and happy (ADHD < Controls)
Seymour 2015 [102]	25 ADHD; 25 controls	Children and adolescents	Emo Go/no-Go (CANTAB)	Commission errors: ADHD > Controls. ADHD made more errors on negative vs positive words compared to controls and showed a bias toward positive emotional stimuli. RT: no group differences
Seymour 2013 [103]	38 ADHD; 41 controls	Children and adolescents	DANVA	Errors: ADHD > Controls for total and fearful child faces. No group differences for adult faces
Shin 2008 [20]	42 ADHD; 27 controls	Children and adolescents	Emotion Recognition Test	Facial emotion recognition: no group differences Contextual understanding score: ADHD < Controls
Sinzig 2008 [104]	30 ADHD; 29 controls	Children and adolescents	Facial affect recognition (FEFA)	Total score faces and eyes: ADHD < Controls. Significant effect for joy (eyes)
Sjöwall 2013 [11]	102 ADHD; 102 controls	Children	Facial emotion recognition	Scores: ADHD < Controls for anger, sadness, fear, happiness, and surprise recognition. No sex differences. Emotion regulation and emotion recognition showed independent effects beyond neuropsychological impairment
Sjöwall 2019 [105]	52 ADHD; 72 controls	Children	Emotion Recognition Task	Errors: ADHD > Controls
Taskiran 2017 [106]	28 ADHD; 20 Controls	Children	Emotion recognition (pictures)	Valence and arousal ratings: No group differences. ADHD with emotion dysregulation (ED) rated unpleasant stimuli as more negative than ADHD without ED
Tatar 2015 [24]	40 ADHD; 40 controls	Adults	POFA	Accuracy: ADHD < Controls for overall outcome and neutral expressions. No difference group in Benton Test. In ADHD group, CPT commissions were associated with erroneously identified emotions and the error rate identifying anger and fear
Tatar 2020 [23]	40 ADHD; 40 controls	Adults	RMET	Correct answers: ADHD < Controls. Mental flexibility measured with the TMT-B predicted performance on the RMET
Tehrani-Doost 2016 [22]	28 ADHD; 27 controls	Children	Facial emotion recognition	Accuracy: ADHD < Controls for anger, happiness, and sadness. No group differences for neutral faces. RT: ADHD > Controls only for happiness
Thoma 2020 [107]	19 ADHD; 20 controls	Adults	TAS-20	Scores: ADHD > Controls, indicating difficulties identifying and describing feelings
Thoma 2020 [108]	19 ADHD; 25 controls	Adults	TAS-20 & RMET	TAS-20 scores: ADHD > Controls. RMET: no group differences
Van Cauwenberge 2015 [109]	29 ADHD; 38 controls	Children and adolescents	SAM rating pictures	Arousal and valence ratings: no group differences RT Emotional n-back: ADHD > Controls
Vetter 2018 [110]	25 ADHD; 25 controls	Children and adolescents	Perceptual discrimination task	RT: no group differences Accuracy: ADHD < Controls
Viering 2021 [111]	61 ADHD; 51 controls	Adolescents and adults	Facial emotion match	RT: ADHD > Controls. Accuracy: ADHD < Controls No group differences in non-emotional condition
Villemonteix 2017 [112]	33 ADHD; 24 controls	Children	Emotional n-back	Accuracy: ADHD < Controls RT: ADHD > Controls in the presence of negative distractors
Walter 2023 [113]	52 ADHD; 24 controls	Adults	Emotional Word Fluency Test	No differences group
Yuill 2007 [114]	19 ADHD; 19 controls	Children	Emotion matching task	Emotional situation-matching: ADHD < Controls for all emotions No differences between ADHD with and without ODD
Yuill 2007 [114]	17 ADHD; 13 controls	Children	Emotion matching task (scaffolding)	Emotional situation-matching with scaffolding: ADHD < Controls
Zhu 2021 [115]	30 ADHD; 20 controls	Children and adolescents	Emo-Stroop Task	RT: ADHD > Controls for positive and negative congruent condition and for positive incongruent condition

*Notes*: ADHD, Attention Deficit Hyperactivity Disorder; ADHD-C, Attention Deficit Hyperactivity Disorder, combined subtype; ADHD-I, Attention Deficit Hyperactivity Disorder, inattentive subtype; ADHD-HI, Attention Deficit Hyperactivity Disorder, hyperactive/impulsive subtype; RT; reaction time; RMET, Reading The Mind In The Eyes Test; ER, emotion recognition; NEPSY-II, Developmental Neuropsychological Assessment, second edition; DANVA, Diagnostic Analysis Of Nonverbal Behavior; IAPS, International Affective Picture System; POFA, Pictures of Facial Affect; TAS-20, Toronto Alexitimia Scale; MNTAP, Minnesota Test of Affective Processing; emo-CPT, emotional Continuous Performance Test; BRIEF, Behavior Rating Inventory of Executive Function; GEES, *Govorna emocionalna ekspresija i stavovi*; CASP, Child and Adolescent Social Perception Measure; CANTAB, Cambridge Neuropsychological Test Automated Battery; TMT-B, Trail Making Test, form B; SAM, Self-Assessment Manikin

**Table 2:** Summary of the key characteristics of the included studies

	N	%	Sample size	Mean	Range
Total	80		6191		
ADHD		53	3257	40.2	10–236
Controls		47	2934	36.2	10–128
Participant age				15.9	
Children/adolescents (< 18)	60	77	4766	10.7	4–18
Adults (18 +)	20	23	1425	31.9	18 <
ADHD presentations	42	53			
Inattentive (%)				28.4	0–87.5
Hyperactive/impulsive (%)				6.8	0–100
Combined (%)				64.6	0–100
Male participants (%)	80			72	41.9–100
ADHD medication status	53				0–100
Without medication	17	21			
Washout period	46	58			
Active medication	7	9			
Presence of co-occurring diagnoses (%)	59	74		23.6	0–100
			*Number of observations*		
Emotion processing tasks					
Indirect measures	30	37.5			
Direct measures	55	68.75			
Type of emotional stimuli					
Face	39	48.75	228		
Eye	17	21.25	39		
Scale	3	3.75	4		
Scene	20	25	123		
Voice	10	12.5	35		
Word	4	5	36		
Emotional category					
Overall	55	68.75	100		
Happiness/positive	38	47.5	91		
Negative	15	18.75	43		
Anger	23	28.75	48		
Fear	17	21.25	36		
Disgust	13	16.25	28		
Sadness	20	25	40		
Neutral	22	27.5	57		
Surprise	9	11.25	22		
Outcome measure					
Accuracy/score	67	83.75	259		
Reaction Time	22	27.5	131		
Other	12	15	75		

ADHD, Attention Deficit Hyperactivity Disorder

### Meta-analysis 1: overall emotion processing

**Fig. 2:**
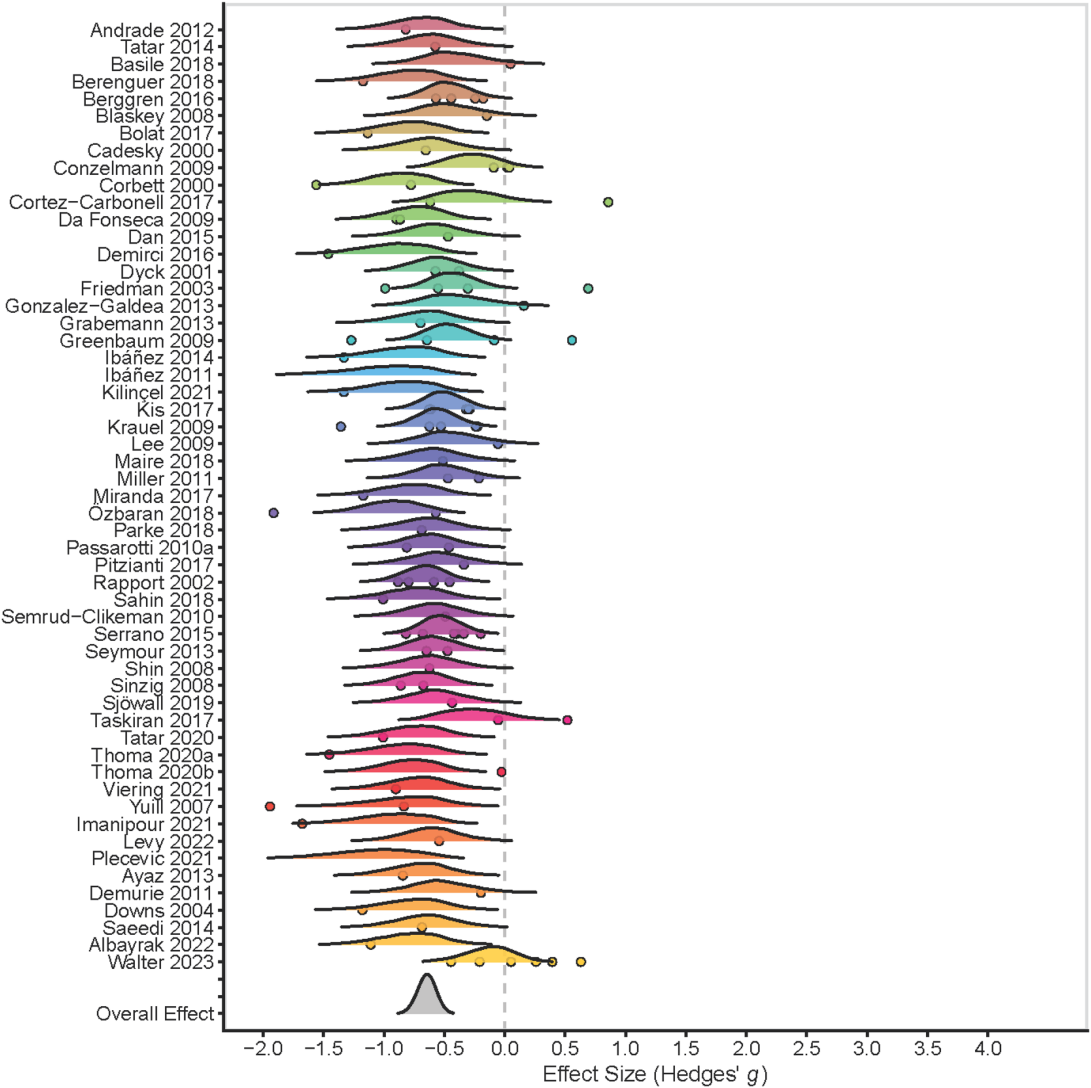
Bayesian forest plot (MA1). Graphs are in different colours to differentiate one study from another. The Bayesian approach allows for an estimation of the full distribution of parameters, rather than providing a point data of average and variability. The curves represent these full distributions of effect sizes. The points within each curve reflect the multilevel nature of the design, where each point is associated with the number of effect sizes included in each study

A summary of the data for the studies included in MA1 is presented in Supplement 4. As shown in Table 3, we found that people with ADHD perform significantly poorer on measures of emotion processing than controls (large effect size). In the assessment of the probability that the parameter is less than zero (i.e., that there really are differences), 100% of its posterior distribution would be compatible with this statement, and the probability of that result with respect to its complementary (parameter greater than zero) is much higher (Bayes Factor >  > 100). In addition, the comparison of the HDI + ROPE regions showed a null overlap between the two, which would allow us to reject the value zero. These results show moderate to high levels of heterogeneity at the within-study level (I^2^ = 48.81%), but low heterogeneity at the between-study level (I^2^ = 33.29%). There was a high publication bias risk (b =  − 2.99, se = 0.93, 95% CrI [-4.55, − 1.47], Credibility = 99%, Evidence Ratio >  > 100). The influence analysis reported no significant results for any effect size. Bayesian forest plot with the distributions of the individual studies is shown in Fig. 2.

**Table 3:** MA1 statistical results

Outcome	*g*	CrI	Within variability	Between variability	Credibility (p < 0)	% overlap HDI + ROPE	ER
Overall	− 0.65	− 0.79, − 0.51	0.31 [0.10, 0.48]	0.41 [0.29, 0.53]	100%	0%	> > 100

CrI, credibility interval; ER, evidence ratio; HDI, high density interval; ROPE, region of practical equivalence

The moderation analyses showed larger effect sizes reported in studies using scales compared to scenes, and using scales compared to faces (see Table 4). This indicates that deficits in emotion processing in ADHD vs controls were more evident in studies using questionnaires/scales compared to those implementing emotional scenes or faces. However, only four studies reported scale outcomes; this probably led to a level of precision below the established threshold (CrI width of scene-scale comparison = 1.19, CrI width of face-scale comparison = 1.04). We did not find any statistically significant effects for other stimuli, i.e., eyes, scenes, and voices, suggesting that these stimuli are similar in detecting differences between people with ADHD and non-psychiatric controls. Lastly, significant differences between the ADHD and control groups were found for all type of stimuli (except words), showing a global emotional processing deficit in ADHD compared to controls.

**Table 4:** Significant moderation effects (MA1)

Moderator	Subtype	Effect size* g*	Contrast	Signification
Type of stimuli	Scenes	− 0.50 (se = 0.12, CrI [− 0.77, − 0.26])		
	Scales	− 1.32 (se = 0.30, CrI [− 1.92, − 0.73])	Scenes > Scales	*b* = 0.82 (se = 0.31, CrI [0.31, 1.33], credibility = 99%, ER > > 100, 0% overlap between HDI-ROPE)
	Faces	− 0.75 (se = 0.31, CrI [− 1.27, − 0.23])	Scales < Faces	*b* = − 0.75 (se = 0.31, CrI [− 1.27, − 0.23], credibility = 99%, ER > 100, 0% overlap between HDI and ROPE)
Outcome measures	Accuracy	− 0.72 (se = 0.06, CrI [− 0.85, − 0.59])		
	RT	− 0.39 (se = 0.15, CrI [− 0.69, − 0.09])	Accuracy < RT	*b* = − 0.33 (se = 0.16, CrI [− 0.60, − 0.06], credibility = 98%, ER = 46.62, 5.8% overlap between HDI and ROPE)
	Other	− 0.08 (se = 0.17, CrI [− 0.33, 0.33])	Accuracy < Other	*b* = − 0.72 (se = 0.17, CrI [− 1.01, − 0.44] credibility = 100%, ER > > 100, 0% overlap between HDI and ROPE)

RT, reaction time; CrI, credibility interval; ER, evidence ratio; HDI, high density interval; ROPE, region of practical equivalence

In terms of the reported outcome measurement, we found larger effect sizes (i.e., differences between ADHD and control groups) for accuracy than RTs, or other outcome measures. However, only for the latter the difference was statistically significant. Specifically, although the 98% of the posterior density distribution supported the presence of the differences between accuracy and RT and despite observing a notable evidence ratio, the HDI and ROPE regions showed an overlap of 5.8%. It should be noted that the precision of the estimations was once again lower than desirable (CrI width of accuracy-RT contrast = 0.54, and CrI width of accuracy-other contrast = 0.57). Moreover, the high magnitude of the differences observed between RTs and Other measures, despite not reaching the significance criterion, is also noteworthy (b = 0.39, se = 0.22, CrI [0.03, 0.75], evidence ratio = 26.62, credibility = 96%, 6.7% overlap HDI-ROPE). Conclusively, this implies that accuracy is the most sensitive outcome measure to identify differences between individuals with ADHD and non-psychiatric controls. The other moderation analyses showed that age, sex, and medication status had no significant effect, indicating that the differences were not due to age, sex nor ADHD medication intake. Due to the heterogeneity of the collected data on comorbidity and ADHD symptom severity, they could not be used in the moderation analyses.

### Meta-analysis 2: specific emotion processing

**Fig. 3:**
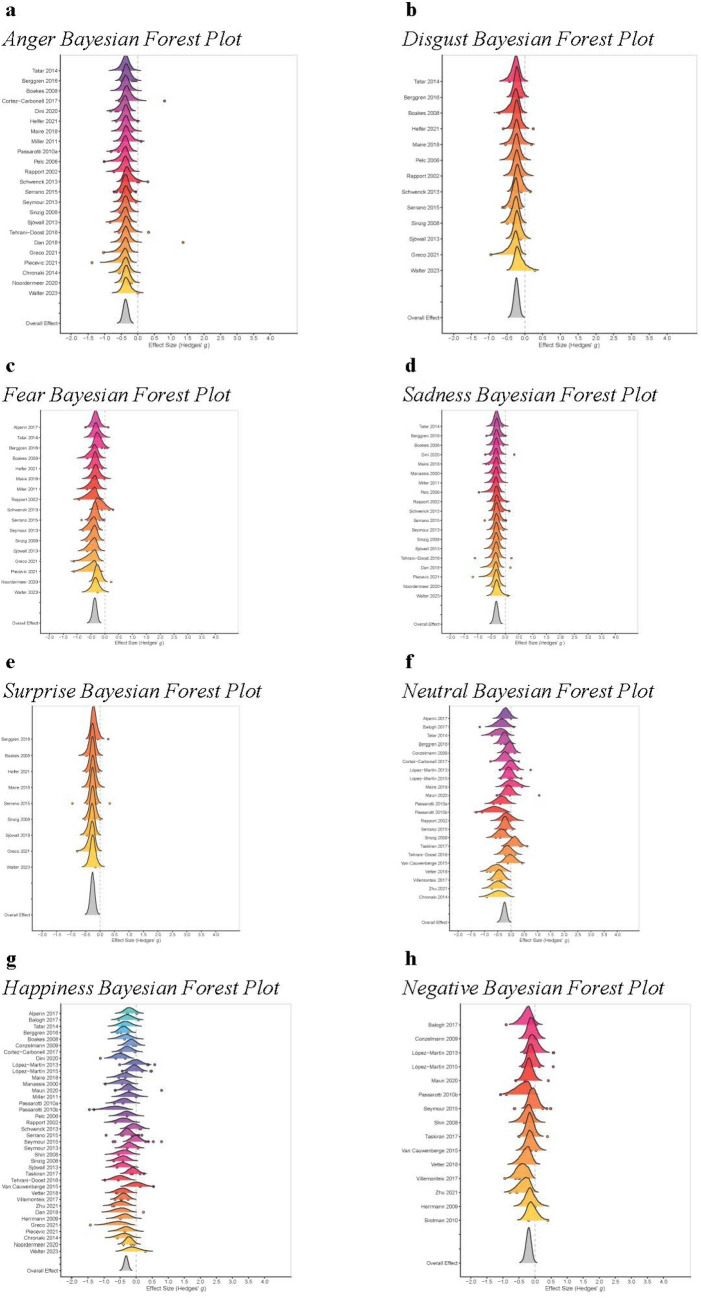
Bayesian forest plot (MA2). Graphs are in different colours to differentiate one study from another. The Bayesian approach allows for an estimation of the full distribution of parameters, rather than providing a point data of average and variability. The curves represent these full distributions of effect sizes. The points within each curve reflect the multilevel nature of the design, where each point is associated with the number of effect sizes included in each study

A description of studies included in MA2 is shown in Supplement 5. MA2 found results in line with MA1 (see Table 5), albeit with smaller effect sizes. Specifically, we found that ADHD participants performed significantly worse on emotion recognition/processing tasks/measures across all emotional categories, except in relation to “negative emotions” (8.2 overlap between the HDI and ROPE regions, nevertheless above the established criterion). This indicates that individuals with ADHD, compared to controls, show a general difficulty in processing emotional cues, regardless of the type of emotion involved. As for MA1, the influence analysis reported no significant results for any effect size, for any discrete emotion. Bayesian forest plots are shown in Fig. 3a–h.

**Table 5:** MA2 statistical results

Outcome	*g*	CrI	Within variability	Between variability	Credibility (*p* < − 0.1)	% overlap HDI + ROPE	ER
Anger	− 0.37	− 0.53, − 0.22	0.12 [0.00, 0.33]	0.37 [0.22, 0.52]	100%	0	> > 100
Disgust	− 0.24	− 0.39, − 0.1	0.12 [0.00, 0.33]	0.13 [0.01, 0.30]	98%	0	39.49
Fear	− 0.37	− 0.54, − 0.22	0.17 [0.01, 0.438]	0.21 [0.05, 0.35]	100%	0	> > 100
Sadness	− 0.34	− 0.49, − 0.19	0.10 [0.00, 0.29]	0.30 [0.14, 0.48]	99%	0	> > 100
Surprise	− 0.26	− 0.43, − 0.11	0.09 [0.00, 0.28]	0.13 [0.01, 0.32]	98%	0	45.08
Happiness/ Positive	− 0.31	− 0.44, − 0.20	0.25 [0.09, 0.39]	0.24 [0.08, 0.37]	100%	0	> > 100
Negative	− 0.20	− 0.38, − 0.04	0.20 [0.02, 0.43]	0.25 [0.05, 0.42]	89%	8.2	8.70
Neutral	− 0.25	− 0.43, − 0.09	0.29 [0.07, 0.48]	0.22 [0.05, 0.39]	97%	0.7	30.75

CrI, credibility interval; ER, evidence ratio; HDI, high density interval; ROPE, region of practical equivalence

Moderation analyses for MA2 showed that *type of stimuli* and *outcome measures* acted as significant moderators of global effect sizes. The use of words as emotional stimuli was associated with more negative effect sizes than other stimuli used in relation to neutral emotions, indicating that neutral words are more difficult to identify as such for people with ADHD vs. controls. Regarding happiness, eyes and face stimuli were associated with more negative effects than scenes. (Table 6), indicating that people with ADHD struggle more to identify positive emotions, compared to controls, when happy faces and eyes stimuli are displayed. Only a few effect sizes could be computed for the word and eyes categories (4 and 3, respectively).

**Table 6:** Significant stimulus type moderators for MA2

Emotion	Stimulus	Effect size* g*	Contrast	Signification
Neutral	Word	− 0.93 (se = 0.26, CrI [− 1.46, − 0.4])		
	Face	− 0.19 (se = 0.10, CrI [− 0.39, 0.02])	Face > Word	*b* = 0.744 (se = 0.29, CrI [1.21, 0.27], credibility = 99.4%, ER > > 100, 0% overlap between HDI-ROPE)
	Eyes	− 0.29, se = 0.24, CrI [− 0.76, 0.19])	Word < Eyes	*b* = − 0.64 (se = 0.36, CrI [− 1.24, − 0.05], credibility = 96%, ER = 26.14, 4.1% overlap between HDI and ROPE)
	Scene	− 0.14 (se = 0.10, CrI [− 0.35, 0.06])	Word < Scene	*b* = − 0.79 (se = 0.29, CrI [− 1.26, − 0.32], credibility = 99.6%, ER > > 100, 0% overlap between HDI and ROPE)
Happiness	Face	− 0.39 (se = 0.08, CrI [− 0.54, − 0.24])		
	Scene	− 0.11 (se = 0.10, CrI [− 0.31, 0.08])	Face < Scene	*b* = − 0.28 (se = 0.12, CrI [− 0.48, − 0.07], credibility = 98.6%, ER = 72.8, 0.05% overlap between HDI and ROPE)
	Eyes	− 0.59 (se = 0.24 CrI [− 1.07, − 0.11])	Eyes < Scene	*b* = − 0.48 (se = 0.26, CrI [− 0.91, − 0.05], credibility = 96.6%, ER > > 100, 0.5% overlap between HDI and ROPE)

CrI, credibility interval; ER, evidence ratio; HDI, high density interval; ROPE, region of practical equivalence

In addition to *type of stimuli*, more negative effect sizes were observed for accuracy than for other measures in relation to both negative emotions and happiness (Table 7). In relation to neutral emotions, a statistically significant difference was observed between accuracy and other measures, and between Accuracy and RTs, with more negative effect sizes for accuracy. This indicates that, at least for negative, neutral and positive emotions, accuracy is more sensitive to detect differences between those with ADHD and non-psychiatric controls, with more difficulties observed in those with ADHD, in line with MA1 results. Finally, in relation to surprise, more negative effect sizes were reported for RTs compared to Other measures. A *post-hoc* comparison across emotional categories was conducted to explore whether differences between ADHD and controls were either equal or different in magnitude depending on emotion. Analysis showed no significant differences, suggesting a global emotion processing deficit in ADHD.

**Table 7:** Significant outcome measure moderators for MA2

Emotion	Measure	Effect size* g*	Contrast	Signification
Negative	Accuracy	− 0.42 (se = 0.13, CrI − 0.68, − 0.17]		
	Other	− 0.07 (se = 0.11, CrI [− 0.31, 0.15]	Accuracy < Other	*b* = − 0.35 (se = 0.16, CrI [− 0.61, − 0.08], credibility = 98%, ER = 60.54, 4% overlap HDI-ROPE)
Neutral	Accuracy	− 0.46 (se = 0.08, CrI [− 0.63, − 0.29]		
	RT	− 0.14 (se = 0.1, CrI [− 0.34, 0.06]	Accuracy < RT	*b* = − 0.32 (se = 0.11, CrI [− 0.5, − 0.14], credibility = 99.6%, ER = > > 100, 0.2% overlap HDI-ROPE)
	Other	0.06 (se = 0.12, CrI [− 0.2, 0.3]	Accuracy < Other	*b* = − 0.52 (se = 0.14, CrI [− 0.75, − 0.28], credibility = 100%, ER = > > 100, 0% overlap HDI-ROPE)
Happiness	Accuracy	− 0.43 (se = 0.07, CrI [− 0.58, − 0.29]		
	Other	− 0.14 (se = 0.10, CrI [− 0.35, 0.07]	Accuracy < Other	*b* = − 0.29 (se = 0.12, CrI [− 0.49, − 0.1], credibility = 99%, ER = > > 100, 3.1% overlap HDI-ROPE)
Suprise	RT	− 0.41, (se = 0.14, CrI [− 0.7, − 0.14]		
	Other	0.12, (se = 0.25, CrI [− 0.39, 0.64]	RT < Other	*b* = 0.53 (se = 0.29, CrI [0.07, 1], credibility = 97%, ER = 31.79, 3.8% overlap HDI-ROPE)

RT, reaction time; CrI, credibility interval; ER, evidence ratio; HDI, high density interval; ROPE, region of practical equivalence

### Sensitivity analysis

Both the estimates of the main effects and those derived from the moderation analyses remained stable irrespective of the prior distribution used (vague or weak) for both MA1 and MA2. See Supplement 6 for more detailed information (Tables S5 to S13).

## Discussion

We conducted a systematic review with Bayesian meta-analysis to meta-analytically determine for the first time whether individuals with ADHD have difficulties in processing emotions, compared to non-psychiatric controls, and to identify what factors may influence these mechanisms. We found evidence of lower accuracy in processing/recognising emotions in people with ADHD, particularly on self-reported questionnaires/scales, supporting the assumption of a global deficit in emotional processing in ADHD. Importantly, we found that individuals with ADHD exhibit difficulties in processing all emotional categories, showing a worse performance regardless of their valence (positive or negative).

To our knowledge, it is the first meta-analysis exploring the effect of the type of stimulus used and the outcome recorded in research comparing emotional processing functioning of individuals with ADHD and non-psychiatric controls. Our results highlight the relevance of taking such variables into account, given that the accuracy measurement, as well as the scales items, seem to be more sensitive in detecting differences between these groups. Our findings are consistent with, and extend, previous meta-analyses conducted on this topic [13, 36]. A general emotion processing deficit in ADHD was observed independently of age, sex, and medication status. Indeed, prior research did not find any effects of sex [11, 30, 65, 91, 101], or age [36] on emotion processing mechanisms in ADHD. Interestingly, medication also did not appear to play a significant role either, although some previous studies [69, 110] found a trend towards normalization of these mechanisms following pharmacological treatment, but this was only tested on small samples. Likewise, a meta-analysis of randomised clinical trials in adults with ADHD suggests a small effect of ADHD medication on the bottom-up mechanisms underlying emotion regulation [116]. Importantly, only 9% of the studies included in our systematic review had participants with ADHD on current medication, while in 60% of the studies a 24–48-h washout period was used.

In MA1, we found that differences between ADHD and control groups on overall emotion processing were more marked when self-reported questionnaires/scales were used, while word stimuli were less sensitive to detect between-group differences. Of note, prior evidence has shown a processing advantage for both emotional scenes and faces over words with affective content [117–119]. It might be that differences between people with ADHD and controls are less evident for those stimuli that elicit less intense emotions (i.e., words). Although there were only three studies using scales, findings based on the Toronto Alexitimia Scale-20 (TAS-20) suggested that people with ADHD may have a lack of self-awareness in their emotional competence [74, 107, 108]. Taken together, these results suggest that there is a global impairment in emotion processing in ADHD affecting emotion recognition, appraisal, and expression. In terms of the outcome measures reported in the studies, we found that accuracy was more sensitive than reaction times or other measures (i.e. arousal, valence and psychophysiological) to detect between-group differences on overall emotional processing. Indeed, most studies found higher accuracy in the control group compared to those with ADHD [11, 24, 57, 62, 81, 105], or no significant differences [31, 32, 71, 77, 83, 93]. No studies found individuals with ADHD performing more accurately than controls. In contrast, results for reaction time (RT) were mixed [19, 66, 85, 88, 97]. Other measures, such as valence and arousal ratings, showed no differences between ADHD and control groups [64, 86, 106, 109], suggesting similar emotional perception intensity.

When emotional processing was examined across the specific emotions in MA2, significant differences were found between ADHD and controls across all emotion categories. Numerous studies have previously reported differences between ADHD and control groups in processing of positive emotions, as assessed by behavioural [11, 54, 62, 66, 68, 97, 104], neural [78, 86] or psychophysiological measures [64]. These differences cannot be attributed to a lack of knowledge or problems retrieving emotional labels, as both groups seem to exhibit similar proficiency in emotional word fluency [113]. Studies that failed to find differences in positive emotions proposed several explanations, such as the potential ceiling effect [59], methodological differences [21], a bias towards positive stimuli [102], and a high variability in emotional responses [53]. Another possible explanation lies in the assumption that positive emotions are seen as a global mood like positive affect or happiness, whereas negative emotions tend to involve a wider range of discrete emotions like anger, fear, sadness, or disgust [120]. In our study, not all discrete negative emotions provide the same differences between people with ADHD and non-psychiatric controls. This could be also happening regarding positive emotion, as Shiota et al. [120] claim in their model of discrete positive emotions. According to this model, the positive dimension would contain a set of discrete emotions each with their neural, cognitive, behavioural, and functional implications, that are based on the neural reward system. Indeed, recent studies have reported differences in the assessment of several positive emotions like awe, contentment, amusement, excitement, serenity, relief, or pleasure [121, 122].

In this second MA, the type of stimuli (i.e., face, eyes, scene, voice and word) and the outcome measures (i.e., accuracy, RT and others) were analysed as moderators of the emotional categories processing. In terms of the type of stimuli, faces were the stimuli that best discriminate between the ADHD and control groups. However, it should be noted that this type of stimulus is the most common in emotional processing research. An important limitation of existing research is that some emotional categories do not include all the types of stimuli considered (e.g., disgust only includes a register of words and does not include voice). In line with MA1, the moderator outcome measures yielded similar results, with accuracy being associated with larger effect sizes than other outcome measures. This was especially true for happiness, negative and neutral categories. Despite reporting the same tasks, accuracy is more sensitive than RT and other outcome measures in detecting between-group differences in emotional processing. Results related to type of stimuli and outcome measures moderators are more controversial, with the reviewed literature showing greater heterogeneity. When assessing emotional processing, laboratory tasks are commonly used, which differ greatly from ecological contexts. Thus, our results are probably underestimating the actual emotional processing impairment in ADHD. For example, Basile et al. [31] found no significant differences between the groups in emotion recognition performance, but they noted that easy items were intentionally selected. However, in more complex tasks involving social scenes, individuals with ADHD identified fewer relevant cues compared to controls [54, 100]. In this regard, Friedman et al. [74] found that adults with ADHD used less emotional vocabulary to describe interactions between two characters they viewed in a film. However, ADHD group did not differ from the control group in their use of non-emotional vocabulary to describe the scenes, suggesting a specific difficulty in emotional functioning. When faced with a dynamic emotion recognition task, ADHD also exhibited more errors and a greater tendency to confuse emotions than controls [76].

We observed that inattention was linked to a higher number of errors in people with ADHD during emotion recognition tasks [89], and it has been suggested that this symptomatologic domain might underlie failures in emotion processing [69], resulting in missing emotional cues. Nevertheless, some studies have not found differences between ADHD and control groups in attentional tasks unrelated to emotion recognition, such as face recognition [24, 74], gender recognition [77], geometric recognition [21], or object recognition tasks [26], so emotional processing differences could not be fully explained by inattention. Conversely, impulsivity can lead to hurried identification based on incomplete data, potentially resulting in misinterpretation of emotions and maladaptive regulatory responses, which are common in ADHD [13, 14, 35]. Even though it remains unclear how core symptoms of ADHD are related to impairments in emotional processing, our results suggest that, despite the high variability in task performance among individuals with ADHD probably due to fluctuations in attention focus, the general difficulty in emotion processing extends beyond the core symptoms of the disorder and cannot be completely explained by them.

Overall, the results of our study highlight the relevance of emotional processing assessment in individuals with ADHD in clinical practice, as this appears to be a critical feature of the disorder. The emotional difficulties observed go beyond the ADHD core symptoms and pharmacological treatment does not seem to have a relevant effect on this regard, hence the need to address this aspect specifically to impact on social relationships and quality of life for people with ADHD.

The findings of this study should be considered in the light of some limitations. Studies in which emotional stimuli have been used in different ways were analysed jointly. While we have found information that converges into robust evidence, further research is needed regarding the complexity of emotional stimuli in ecological contexts. Furthermore, due to limitations in funding, we limited the search to articles English language. Despite potential methodological limitations that may exclude relevant studies, this study's extensive inclusion of papers and use of Bayesian methodology ensure robust results. Future research should explore ADHD's impact on emotion processing using dynamic tasks resembling real-life interactions, across different time points and while controlling for attention, impulsivity, and symptom severity. It remains uncertain whether the observed emotion processing deficits in our study are primary or secondary to attentional and executive function impairments in ADHD. While some suggest these deficits relate to working memory failures in ADHD [123, 124], further research is needed. Additionally, investigating positive emotions in ADHD may shed light on variability in results in this area.

## Conclusions

This study indicates that individuals with ADHD show impairments in recognizing and processing emotions, which appear consistent across age, sex, and pharmacological conditions. These impairments span all basic emotions, suggesting a widespread deficit with notable variability. Therefore, assessing emotion processing in ADHD using composite scores across various ecological contexts and time points could help establish a specific profile for improved detection and diagnosis in clinical practice.

## Supplementary Information

Below is the link to the electronic supplementary material.

Supplementary file1 (DOCX 84 kb)

## Data Availability

Data is provided within the manuscript or supplementary information files.
