# Supplementary material for: Emotion processing difficulties in ADHD: a Bayesian meta-analysis study
Source: Eur Child Adolesc Psychiatry. 2025 Jan 24;34(8):2369–90. doi: 10.1007/s00787-025-02647-3 (PMC12397143; doi:10.1007/s00787-025-02647-3)
Supplement: Supplementary file 1 — Supplementary file1 (DOCX 84 kb) [file 787_2025_2647_MOESM1_ESM.docx]

**Emotion processing difficulties in ADHD: a Bayesian meta-analysis study**

Ana-María Soler-Gutiérrez ^1,2^, Alberto J. Sánchez-Carmona ^3^, Jacobo Albert ^4^, José Antonio Hinojosa ^5-7^, Samuele Cortese ^8-13^, Alessio Bellato* ^8,9,14-16^ and Julia Mayas* ^1^

^1^Faculty of Psychology, Universidad Nacional de Educación a Distancia (UNED), Madrid, Spain

^2^Escuela Internacional de Doctorado de la UNED (EIDUNED), Universidad Nacional de Educación a Distancia (UNED), Madrid, Spain

^3^Centro Neuromottiva, 28016, Madrid, Spain

^4^Faculty of Psychology, Universidad Autónoma de Madrid, Madrid, Spain

^5^Instituto Pluridisciplinar, Universidad Complutense de Madrid, Madrid, Spain

^6^Faculty of Psychology, Universidad Complutense de Madrid, Madrid, Spain

^7^Centro de Investigación Nebrija en Cognición (CINC), Universidad Nebrija, Madrid, Spain

^8^School of Psychology, University of Southampton, Southampton, United Kingdom

^9^Centre for Innovation in Mental Health, University of Southampton, Southampton, United Kingdom

^10^Solent NHS Trust, Southampton, UK

^11^Clinical and Experimental Sciences (CNS and Psychiatry), Faculty of Medicine, University of Southampton, Southampton, UK.

^12^Hassenfeld Children's Hospital at NYU Langone, New York University Child Study Center, New York, NY, USA

^13^DiMePRe-J - Department of Precision and Rigenerative Medicine-Jonic Area, Università degli Studi di Bari “Aldo Moro”, Bari, Italy

^14^Institute for Life Sciences, University of Southampton, Southampton, United Kingdom

^15^School of Psychology, University of Nottingham, Semenyih, Malaysia

^16^Mind and Neurodevelopment (MiND) Research Group, University of Nottingham, Semenyih, Malaysia

Corresponding author: Julia Mayas, jmayas@psi.uned.es

# Supplementary Information

[Supplementary Information 3](#_Toc170234083)

[Supplement 1. Table S1. PRISMA Checklist 4](#_Toc170234084)

[Supplement 2. Search strategy 9](#_Toc170234085)

[Supplement 3. Table S2. Appraisal tool for Cross-Sectional Studies (AXIS) 11](#_Toc170234086)

[Supplement 4. Table S3. Descriptive data of the articles included in MA 1. 16](#_Toc170234087)

[Supplement 5. Table S4. Descriptive data of the articles included in MA 2. 17](#_Toc170234088)

[Supplement 6. Tables S5 to S13. Sensitivity analysis 19](#_Toc170234089)

# Supplement 1. Table S1. PRISMA Checklist

| **Section and Topic** | **Item #** | **Checklist item** | **Location where item is reported** |
| --- | --- | --- | --- |
| **TITLE** | | |  |
| Title | 1 | Identify the report as a systematic review. | Title Page |
| **ABSTRACT** | | |  |
| Abstract | 2 | See the PRISMA 2020 for Abstracts checklist. | Abstract |
| **INTRODUCTION** | | |  |
| Rationale | 3 | Describe the rationale for the review in the context of existing knowledge. | Introduction |
| Objectives | 4 | Provide an explicit statement of the objective(s) or question(s) the review addresses. | Introduction |
| **METHODS** | | |  |
| Eligibility criteria | 5 | Specify the inclusion and exclusion criteria for the review and how studies were grouped for the syntheses. | Methods, paragraph “Selection criteria” |
| Information sources | 6 | Specify all databases, registers, websites, organisations, reference lists and other sources searched or consulted to identify studies. Specify the date when each source was last searched or consulted. | Methods, paragraph “Search strategy” |
| Search strategy | 7 | Present the full search strategies for all databases, registers and websites, including any filters and limits used. | Supplement 2 |
| Selection process | 8 | Specify the methods used to decide whether a study met the inclusion criteria of the review, including how many reviewers screened each record and each report retrieved, whether they worked independently, and if applicable, details of automation tools used in the process. | Methods, paragraph “Data selection, extraction and coding” |
| Data collection process | 9 | Specify the methods used to collect data from reports, including how many reviewers collected data from each report, whether they worked independently, any processes for obtaining or confirming data from study investigators, and if applicable, details of automation tools used in the process. | Methods, paragraph “Data selection, extraction and coding” |
| Data items | 10a | List and define all outcomes for which data were sought. Specify whether all results that were compatible with each outcome domain in each study were sought (e.g. for all measures, time points, analyses), and if not, the methods used to decide which results to collect. | Methods, paragraphs “Data selection, extraction and coding” and “Outcomes and assessment of study quality” |
|  | 10b | List and define all other variables for which data were sought (e.g. participant and intervention characteristics, funding sources). Describe any assumptions made about any missing or unclear information. | Methods, paragraph “Data selection, extraction and coding” |
| Study risk of bias assessment | 11 | Specify the methods used to assess risk of bias in the included studies, including details of the tool(s) used, how many reviewers assessed each study and whether they worked independently, and if applicable, details of automation tools used in the process. | Methods, paragraph “Outcomes and assessment of study quality” and Supplement 3 |
| Effect measures | 12 | Specify for each outcome the effect measure(s) (e.g. risk ratio, mean difference) used in the synthesis or presentation of results. | Methods, paragraph “Data synthesis and analysis” |
| Synthesis methods | 13a | Describe the processes used to decide which studies were eligible for each synthesis (e.g. tabulating the study intervention characteristics and comparing against the planned groups for each synthesis (item #5)). | Methods, paragraph “Data synthesis and analysis” |
|  | 13b | Describe any methods required to prepare the data for presentation or synthesis, such as handling of missing summary statistics, or data conversions. | Methods, paragraph “Data synthesis and analysis” |
|  | 13c | Describe any methods used to tabulate or visually display results of individual studies and syntheses. | Methods, paragraph “Data synthesis and analysis” |
|  | 13d | Describe any methods used to synthesize results and provide a rationale for the choice(s). If meta-analysis was performed, describe the model(s), method(s) to identify the presence and extent of statistical heterogeneity, and software package(s) used. | Methods, paragraph “Data synthesis and analysis” |
|  | 13e | Describe any methods used to explore possible causes of heterogeneity among study results (e.g. subgroup analysis, meta-regression). | Methods, paragraph “Data synthesis and analysis” |
|  | 13f | Describe any sensitivity analyses conducted to assess robustness of the synthesized results. | Methods, paragraph “Data synthesis and analysis” |
| Reporting bias assessment | 14 | Describe any methods used to assess risk of bias due to missing results in a synthesis (arising from reporting biases). | Methods, paragraph “Outcomes and assessment of study quality” and Supplement 3 |
| Certainty assessment | 15 | Describe any methods used to assess certainty (or confidence) in the body of evidence for an outcome. | n/a |
| **RESULTS** | | |  |
| Study selection | 16a | Describe the results of the search and selection process, from the number of records identified in the search to the number of studies included in the review, ideally using a flow diagram. | Results and Figure 1 |
|  | 16b | Cite studies that might appear to meet the inclusion criteria, but which were excluded, and explain why they were excluded. | n/a |
| Study characteristics | 17 | Cite each included study and present its characteristics. | Table 1 |
| Risk of bias in studies | 18 | Present assessments of risk of bias for each included study. | Supplement 3 |
| Results of individual studies | 19 | For all outcomes, present, for each study: (a) summary statistics for each group (where appropriate) and (b) an effect estimate and its precision (e.g. confidence/credible interval), ideally using structured tables or plots. | Tables 2, 3 and 5; Figures 2 and 3 |
| Results of syntheses | 20a | For each synthesis, briefly summarise the characteristics and risk of bias among contributing studies. | Supplement 3, 4 and 5 |
|  | 20b | Present results of all statistical syntheses conducted. If meta-analysis was done, present for each the summary estimate and its precision (e.g. confidence/credible interval) and measures of statistical heterogeneity. If comparing groups, describe the direction of the effect. | Results, Tables 3 and 4, Figure 2 and 3, Supplement 6 |
|  | 20c | Present results of all investigations of possible causes of heterogeneity among study results. | Results, Figure 2, Supplements 4, 5 and 6 |
|  | 20d | Present results of all sensitivity analyses conducted to assess the robustness of the synthesized results. | Results paragraph “sensitivity analysis” and Suplement 6 |
| Reporting biases | 21 | Present assessments of risk of bias due to missing results (arising from reporting biases) for each synthesis assessed. | n/a |
| Certainty of evidence | 22 | Present assessments of certainty (or confidence) in the body of evidence for each outcome assessed. | n/a |
| **DISCUSSION** | | |  |
| Discussion | 23a | Provide a general interpretation of the results in the context of other evidence. | Discussion, paragraphs 1, 2 |
|  | 23b | Discuss any limitations of the evidence included in the review. | Discussion |
|  | 23c | Discuss any limitations of the review processes used. | Discussion, paragraph 8 |
|  | 23d | Discuss implications of the results for practice, policy, and future research. | Discussion, paragraphs 8, 9 |
| **OTHER INFORMATION** | | |  |
| Registration and protocol | 24a | Provide registration information for the review, including register name and registration number, or state that the review was not registered. | Methods |
|  | 24b | Indicate where the review protocol can be accessed, or state that a protocol was not prepared. | Methods |
|  | 24c | Describe and explain any amendments to information provided at registration or in the protocol. | n/a |
| Support | 25 | Describe sources of financial or non-financial support for the review, and the role of the funders or sponsors in the review. | Title Page, Abstract Page |
| Competing interests | 26 | Declare any competing interests of review authors. | Abstract Page |
| Availability of data, code and other materials | 27 | Report which of the following are publicly available and where they can be found: template data collection forms; data extracted from included studies; data used for all analyses; analytic code; any other materials used in the review. | Methods, paragraph “Data synthesis and analysis” |

*From:*  Page MJ, McKenzie JE, Bossuyt PM, Boutron I, Hoffmann TC, Mulrow CD, et al. The PRISMA 2020 statement: an updated guideline for reporting systematic reviews. BMJ 2021;372:n71. doi: 10.1136/bmj.n71

# Supplement 2. Search strategy

**Last Search Date: 3^rd^ December 2023**

EBSCOhost (MEDLINE, ERIC, PSYCINFO) results: 543

Line 1 Title: ADHD OR ADD OR “attention deficit hyperactivity disorder” OR “attention deficit disorder” OR “hyperkinetic disorder”

Line 2 Title: emotion* OR labil* OR affect* OR negative* OR irritability OR frustration OR “theory of mind” OR empathy

Filters: Publication date since 1998, Academic publications, Empirical studies, English

SCOPUS results: 922

Line 1 Title: ADHD OR ADD OR “attention deficit hyperactivity disorder” OR “attention deficit disorder” OR “hyperkinetic disorder”

Line 2 Title: emotion* OR labil* OR affect* OR negative* OR irritability OR frustration OR “theory of mind” OR empathy

Limited to: Publication date since 1998, Articles, English

WOS results: 1.311

Line 1 Title: ADHD OR ADD OR “attention deficit hyperactivity disorder” OR “attention deficit disorder” OR “hyperkinetic disorder”

Line 2 Title: emotion* OR labil* OR affect* OR negative* OR irritability OR frustration OR “theory of mind” OR empathy

Limited to: Publication date since 1998, Articles, English

TOTAL = 2.776

Searching for and removing duplicates: 1.413

Records screened: 1.363

SELECTED PAPERS FOR FULL TEXT REVIEW: 144

Papers excluded and reason (n = 81):

1. No DSM/ICD ADHD diagnosis reported: 14
2. No useful or reported ER data: 62
3. No typical developmental control group: 3
4. No pure ADHD group: 1
5. PDF no available: 1

Manually retrieved: 17

**INCLUDED PAPERS: 80**

# Supplement 3. Table S2. Appraisal tool for Cross-Sectional Studies (AXIS)

| **1^st^ author and Year** | **Clear aims** | **Appropriate study design** | **Justified sample size** | **Clear target population** | **Representative sample** | **Appropriate selection process** | **Appropriate categorization of non-responders** | **Appropriate measurement of risk factors and outcome** | **Appropriate measurments** | **Appropriate determination of statistical significance** | **Clear description of methods** | **Adequate description of basic data** | **Absence of non-response bias** | **Appropriate description of non-responders** | **Consistency of results** | **Presentation of results for all analyses described in methods** | **Justified discussions and conclusions** | **Discussion of limitations of the study** | **Absence of conflict of interest** | **Ethical approval and consent** |
| --- | --- | --- | --- | --- | --- | --- | --- | --- | --- | --- | --- | --- | --- | --- | --- | --- | --- | --- | --- | --- |
| Albayrak 2022 | Y | Y | N | Y | N | Y | Y | Y | Y | Y | Y | Y | N | N | Y | Y | Y | Y | Y | Y |
| Alperin 2017 | Y | Y | N | Y | Y | Y | N | Y | Y | Y | Y | Y | N | N | Y | Y | Y | N | Y | Y |
| Andrade 2012 | Y | Y | N | Y | Y | Y | N | Y | Y | Y | Y | Y | N | N | Y | Y | Y | Y | Y | Y |
| Ayaz 2013 | Y | Y | N | Y | Y | Y | N | Y | Y | Y | Y | Y | N | N | Y | Y | Y | Y | N | Y |
| Balogh 2017 | Y | Y | N | Y | Y | Y | N | Y | Y | Y | Y | Y | N | N | Y | Y | Y | Y | Y | Y |
| Basile 2018 | Y | Y | N | Y | Y | Y | N | Y | Y | Y | Y | Y | N | Y | Y | Y | Y | Y | Y | Y |
| Berenguer 2018 | Y | Y | N | Y | Y | Y | N | Y | Y | Y | Y | Y | N | N | Y | Y | Y | Y | Y | Y |
| Berggren 2016 | Y | Y | N | Y | Y | Y | Y | Y | Y | Y | Y | Y | N | Y | Y | Y | Y | Y | Y | Y |
| Blaskey 2008 | Y | Y | N | Y | Y | Y | N | Y | Y | Y | Y | Y | N | Y | Y | Y | Y | N | N | N |
| Boakes 2008 | Y | Y | N | Y | N | Y | N | Y | Y | N | Y | Y | N | N | Y | Y | Y | N | N | N |
| Bolat 2017 | N | Y | N | Y | Y | Y | N | Y | Y | Y | Y | N | N | N | Y | Y | Y | Y | N | Y |
| Brotman 2010 | Y | Y | N | Y | Y | Y | N | Y | Y | Y | Y | Y | N | N | Y | Y | Y | Y | Y | Y |
| Cadesky 2000 | Y | Y | N | Y | Y | Y | N | Y | Y | Y | Y | N | N | N | Y | Y | Y | Y | N | N (CONSENT) |
| Chronaki 2014 | Y | Y | N | Y | N | Y | N | Y | Y | Y | Y | N | N | N | Y | Y | Y | Y | Y | Y |
| Conzelmann 2009 | Y | Y | N | Y | Y | Y | N | Y | Y | Y | Y | Y | N | Y | Y | Y | Y | Y | Y | N (CONSENT) |
| Corbett 2000 | Y | Y | Y | Y | N | Y | N | Y | Y | Y | Y | Y | N | N | Y | Y | Y | N | N | Y |
| Cortez-Carbonell 2017 | Y | Y | N | Y | Y | Y | N | Y | Y | Y | Y | Y | N | N | Y | Y | Y | Y | Y | N (CONSENT) |
| Da Fonseca 2009 | Y | Y | N | Y | N | Y | N | Y | Y | Y | Y | Y | N | N | Y | Y | Y | N | N | Y |
| Dan 2018 | Y | Y | N | Y | N | Y | N | Y | Y | Y | Y | Y | N | N | Y | Y | Y | Y | Y | Y |
| Dan 2015 | Y | Y | N | Y | N | Y | N | Y | Y | Y | Y | Y | N | N | Y | Y | Y | Y | Y | Y |
| Demirci 2016 | Y | Y | N | Y | Y | Y | Y | Y | Y | Y | Y | Y | N | Y | Y | Y | Y | Y | Y | Y |
| Demurie 2011 | Y | Y | N | Y | N | Y | Y | Y | Y | Y | Y | N | N | N | Y | Y | Y | Y | N | Y |
| Dini 2020 | Y | Y | Y | Y | N | Y | N | Y | Y | Y | Y | Y | N | N | Y | Y | Y | Y | N | N |
| Downs 2004 | Y | Y | N | Y | N | Y | N | Y | Y | Y | Y | N | N | N | Y | Y | Y | Y | N | N (CONSENT) |
| Dyck 2001 | Y | Y | N | Y | Y | Y | Y | Y | Y | Y | Y | Y | N | Y | Y | Y | Y | N | N | N |
| Friedman 2003 | Y | Y | N | Y | Y | Y | Y | Y | Y | Y | Y | Y | N | N | Y | Y | Y | Y | N | Y |
| Gonzalez-Gadea 2013 | Y | Y | N | Y | Y | Y | N | Y | Y | Y | Y | Y | N | N | Y | Y | Y | Y | Y | Y |
| Grabemann 2013 | Y | Y | N | Y | N | Y | N | Y | Y | Y | Y | Y | N | N | Y | Y | Y | Y | N | Y |
| Greco 2021 | Y | Y | N | Y | N | Y | N | Y | Y | Y | Y | N | N | N | Y | Y | Y | Y | Y | Y |
| Greenbaum 2009 | Y | Y | N | Y | Y | Y | Y | Y | Y | Y | Y | Y | N | N | Y | Y | Y | Y | N | Y |
| Helfer 2021 | Y | Y | N | Y | Y | Y | N | Y | Y | Y | Y | Y | N | N | Y | Y | Y | Y | Y | Y |
| Herrmann 2009 | Y | Y | N | Y | N | Y | N | Y | Y | Y | Y | N | N | N | Y | Y | Y | Y | Y | Y |
| Ibáñez 2014 | Y | Y | N | Y | N | Y | N | Y | Y | Y | Y | Y | N | N | Y | Y | Y | Y | N | Y |
| Ibáñez 2011 | Y | Y | N | Y | N | Y | N | Y | Y | Y | Y | Y | N | N | Y | Y | Y | Y | Y | Y |
| Imanipour 2021 | Y | Y | N | Y | Y | Y | N | Y | Y | Y | Y | N | N | N | Y | Y | Y | Y | Y | Y |
| Kılınçel 2021 | Y | Y | N | Y | Y | Y | Y | Y | Y | Y | Y | Y | N | N | Y | Y | Y | Y | Y | Y |
| Kis 2017 | Y | Y | N | Y | Y | Y | N | Y | Y | Y | Y | Y | N | N | Y | Y | Y | N | Y | Y |
| Krauel 2009 | Y | Y | N | Y | N | Y | N | Y | N | Y | Y | Y | N | N | Y | Y | Y | N | N | Y |
| Lee 2009 | Y | Y | Y | Y | Y | Y | Y | Y | Y | Y | Y | Y | N | Y | Y | Y | Y | Y | N | N (CONSENT) |
| Levy 2022 | Y | Y | N | Y | Y | Y | Y | Y | Y | Y | Y | Y | N | Y | Y | Y | Y | Y | Y | Y |
| López-Martín 2013 | Y | Y | N | Y | N | Y | N | Y | Y | Y | Y | Y | N | N | Y | Y | N | N | N | Y |
| López-Martín 2015 | Y | Y | N | Y | Y | Y | N | Y | Y | Y | Y | Y | N | N | Y | Y | Y | Y | Y | Y |
| Maire 2018 | Y | Y | N | Y | Y | Y | N | Y | Y | Y | Y | Y | N | Y | Y | Y | Y | Y | Y | Y |
| Manassis 2000 | Y | Y | N | Y | N | Y | N | Y | Y | Y | Y | Y | N | N | Y | Y | Y | Y | N | Y |
| Mauri 2020 | Y | Y | N | Y | Y | Y | Y | Y | N | Y | Y | Y | N | Y | Y | Y | Y | Y | Y | Y |
| Miller 2011 | Y | Y | N | Y | N | Y | Y | Y | Y | Y | Y | Y | N | Y | Y | Y | Y | Y | N | Y |
| Miranda 2017 | Y | Y | N | Y | Y | Y | N | Y | Y | Y | Y | Y | N | N | Y | Y | Y | Y | Y | Y |
| Noordermeer 2020 | Y | Y | N | Y | N | Y | N | Y | Y | Y | Y | Y | N | N | Y | Y | Y | Y | Y | Y |
| Özbaran 2018 | Y | Y | N | Y | Y | Y | Y | Y | Y | Y | Y | Y | N | Y | Y | Y | Y | Y | Y | Y |
| Parke 2018 | Y | Y | N | Y | Y | Y | N | Y | Y | Y | Y | Y | N | N | Y | Y | Y | Y | Y | N |
| Passarotti 2010 | Y | Y | N | Y | N | Y | N | Y | Y | Y | Y | Y | N | N | Y | Y | Y | Y | Y | Y |
| Passarotti 2010 | Y | Y | N | Y | N | Y | N | Y | Y | Y | Y | Y | N | N | Y | Y | Y | Y | Y | Y |
| Pelc 2006 | Y | Y | N | Y | N | Y | N | Y | Y | Y | Y | N | N | N | Y | Y | Y | N | N | Y |
| Pitzianti 2017 | Y | Y | N | Y | Y | Y | N | Y | Y | Y | Y | Y | N | N | Y | Y | Y | N | Y | N (CONSENT) |
| Plecevic 2021 | Y | Y | N | Y | Y | Y | N | Y | Y | Y | Y | N | N | N | Y | Y | Y | Y | Y | Y |
| Rapport 2002 | Y | Y | N | Y | Y | Y | N | Y | Y | Y | Y | Y | N | N | Y | Y | Y | Y | N | N (CONSENT) |
| Saeedi 2014 | Y | Y | N | Y | N | Y | N | Y | Y | Y | Y | N | N | N | Y | Y | Y | Y | Y | Y |
| Sahin 2018 | Y | Y | N | Y | Y | Y | Y | Y | Y | Y | Y | Y | N | Y | Y | Y | Y | Y | N | Y |
| Schwenck 2013 | Y | Y | N | Y | Y | Y | N | Y | Y | Y | Y | Y | N | N | Y | Y | Y | Y | N | Y |
| Semrud-Clikeman 2010 | Y | Y | N | Y | Y | Y | Y | Y | Y | Y | Y | Y | N | Y | Y | Y | Y | Y | N | N |
| Serrano 2015 | Y | Y | N | Y | Y | Y | N | Y | Y | N | Y | Y | N | N | Y | Y | Y | Y | Y | Y |
| Seymour 2015 | Y | Y | N | Y | Y | Y | N | Y | Y | Y | Y | Y | N | N | Y | Y | Y | Y | Y | Y |
| Seymour 2013 | Y | Y | N | Y | Y | Y | N | Y | Y | Y | Y | Y | N | N | Y | Y | Y | Y | Y | Y |
| Shin 2008 | Y | Y | N | Y | N | Y | N | Y | Y | Y | Y | Y | N | N | Y | Y | Y | Y | N | N |
| Sinzig 2008 | Y | Y | N | Y | Y | Y | N | Y | Y | Y | Y | Y | N | N | Y | Y | Y | Y | N | N (CONSENT) |
| Sjöwall 2013 | Y | Y | N | Y | Y | Y | N | Y | Y | Y | Y | N | N | N | Y | Y | Y | Y | Y | Y |
| Sjöwall 2019 | Y | Y | N | Y | Y | Y | N | Y | Y | Y | Y | Y | N | N | Y | Y | Y | Y | Y | Y |
| Taskiran 2017 | Y | Y | N | Y | Y | Y | N | Y | Y | Y | Y | Y | N | N | Y | Y | Y | Y | Y | Y |
| Tatar 2015 | Y | Y | N | Y | Y | Y | Y | Y | Y | Y | Y | Y | N | N | Y | Y | Y | Y | N | Y |
| Tatar 2020 | Y | Y | N | Y | Y | Y | Y | Y | Y | Y | Y | Y | N | N | Y | Y | Y | Y | Y | Y |
| Tehrani-Doost 2016 | Y | Y | N | Y | N | Y | N | Y | Y | Y | Y | Y | N | N | Y | Y | Y | Y | Y | N |
| Thoma 2020 | Y | Y | N | Y | N | Y | N | Y | Y | Y | Y | Y | N | N | Y | Y | Y | Y | Y | Y |
| Thoma 2020 | Y | Y | N | Y | N | Y | Y | Y | Y | Y | Y | Y | N | Y | Y | Y | Y | Y | Y | Y |
| Van Cauwenberge 2015 | Y | Y | N | Y | Y | Y | Y | Y | Y | Y | Y | Y | N | Y | Y | Y | Y | Y | N | Y |
| Vetter 2018 | Y | Y | N | Y | N | Y | Y | Y | Y | Y | Y | Y | N | N | Y | Y | Y | Y | N | Y |
| Viering 2021 | Y | Y | N | Y | Y | Y | Y | Y | Y | Y | Y | Y | N | N | Y | Y | Y | Y | Y | Y |
| Villemonteix 2017 | Y | Y | N | Y | Y | Y | Y | Y | Y | Y | Y | Y | N | N | Y | Y | Y | Y | Y | Y |
| Walter 2023 | Y | Y | N | Y | N | Y | N | Y | Y | Y | Y | Y | N | N | Y | Y | Y | Y | Y | Y |
| Yuill 2007 | Y | Y | N | Y | N | Y | N | Y | Y | Y | Y | N | N | N | Y | Y | Y | Y | N | N |
| Zhu 2021 | Y | Y | Y | Y | Y | Y | Y | Y | Y | Y | Y | Y | N | N | Y | Y | Y | Y | Y | Y |

*From*: Downes, M. J., Brennan, M. L., Williams, H. C., & Dean, R. S. Appraisal tool for Cross-Sectional Studies (AXIS). BMJ Open [Internet]. 2016; 6 (12): 1–7.

# Supplement 4. Table S3. Descriptive data of the articles included in MA 1.

|  | ***N*** | **%** | **Sample size** | **Mean** | **Range** |
| --- | --- | --- | --- | --- | --- |
| Total   - ADHD - Controls | 55 |  | 4635  2481  2154 | 44.3  38.5 | 10 – 236  10 – 128 |
| Participant age   - Children/adolescents (<18) - Adults (18<) | 39  16 | 71  29 | 3434  1201 | 10.3  32.4 | 4-18  18< |
| ADHD presentations   - Inattentive (%) - Hyperactive/impulsive (%) - Combined (%) | 27 | 49 |  | 31.4  4.4  63.8 | 0 – 87.5  0 – 35.5  31.1 – 100 |
| Male participants (%) | 55 | 100 |  | 69.71 | 41.9 – 100 |
| ADHD medication status reported   - Without medication - Washout period - Active medication | 47  13  27  7 | 85.5  27.7  57.4  14.9 |  |  |  |
| Comorbidity reported (%) | 39 | 70.9 |  | 26.5 | 0 – 100 |
|  |  |  | **Observations (total n)** |  |  |
| Emotional processing task   - Indirect measures - Direct measures | 13  45 | 23.6  81.8 |  |  |  |
| Type of stimuli   - Face - Eyes - Scales - Scenes - Voice - Words | 25  17  3  13  8  1 | 45.5  30.9  5.5  23.6  14.5  1.8 | 35  18  4  25  12  6 |  |  |
| Outcome measurement   - Accuracy/score - Reaction time (RT) - Other | 51  7  5 | 92.7  12.7  9 | 76  12  12 |  |  |

Notes: ADHD, Attention Deficit Hyperactivity Disorder.

# Supplement 5. Table S4. Descriptive data of the articles included in MA 2.

|  | ***N*** | **%** | **Sample size** | **Mean** | **Range** |
| --- | --- | --- | --- | --- | --- |
| Total   - ADHD - Controls | 40 |  | 2754  1444  1310 | 36.1  32.8 | 14 – 197  14 – 128 |
| Participant age   - Children/adolescents (<18) - Adults (18<) | 30  10 | 75  25 | 1882  872 | 11.1  31.1 | 6 – 18  18 < |
| ADHD presentations   - Inattentive (%) - Hyperactive/impulsive (%) - Combined (%) | 22 | 55 |  | 28.9  8.8  62.3 | 0 – 87.5  0 – 100  0 – 100 |
| Male (%) | 40 | 100 |  | 73.1 | 47 – 100 |
| ADHD medication status   - Without medication - Washout period - Active medication | 36  9  27  0 | 90  25  75  0 |  |  |  |
| Presence of co-occurring diagnoses (%) | 31 | 77.5 |  | 22.5 | 0 – 77.6 |
|  | | | **Observations (total nº)** |  | |
| Emotional processing task   - Indirect measures - Direct measures | 17  24 | 42.5  60 |  |  |  |
| Type of stimuli   - Face - Eyes - Scenes - Voice - Words | 23  2  10  5  4 | 57.5  5  25  12.5  10 | 193  21  98  23  30 |  |  |
| Emotional category   - Happiness/positive - Negative - Anger - Fear - Disgust - Sadness - Neutral - Surprise | 38  15  23  17  13  20  22  9 | 95  37.5  57.5  42.5  32.5  50  55  22.5 | 91  43  48  36  28  40  57  22 |  |  |
| Outcome measurement   - Accuracy/score - Reaction time (RT) - Other | 31  18  10 | 77.5  45  25 | 183  119  63 |  |  |

Notes: ADHD, Attention Deficit Hyperactivity Disorder.

# Supplement 6. Tables S5 to S13. Sensitivity analysis

**Table S5.** Sensitivity analysis of MA 1

| **Model** | **Outcome** | **Prior** | **Estimate** | **Std.error** | **Conf.low** | **Conf.high** |
| --- | --- | --- | --- | --- | --- | --- |
| Main | Summary effect [global] | Analysis prior | -0,646 | 0,072 | -0,792 | -0,508 |
| Main_vp | Summary effect [global] | Vague prior | -0,647 | 0,073 | -0,794 | -0,508 |
| Main_wp | Summary effect [global] | Weak prior | -0,649 | 0,073 | -0,795 | -0,509 |
| Age | Age [intercept] | Analysis prior | -0,803 | 0,126 | -1,053 | -0,556 |
| Age_vp | Age [intercept] | Vague prior | -0,816 | 0,125 | -1,062 | -0,571 |
| Age_wp | Age [intercept] | Weak prior | -0,817 | 0,127 | -1,066 | -0,57 |
| Sex | Sex [intercept] | Analysis prior | -0,208 | 0,273 | -0,75 | 0,324 |
| Sex_vp | Sex [intercept] | Vague prior | -0,222 | 0,285 | -0,782 | 0,333 |
| Sex_wp | Sex [intercept] | Weak prior | -0,226 | 0,285 | -0,79 | 0,335 |
| Med | Med [intercept] | Analysis prior | -0,547 | 0,189 | -0,92 | -0,18 |
| Med_vp | Med [intercept] | Vague prior | -0,572 | 0,192 | -0,948 | -0,197 |
| Med_wp | Med [intercept] | Weak prior | -0,572 | 0,195 | -0,958 | -0,191 |
| Stimuli | Stimuli [intercept] | Analysis prior | -0,505 | 0,129 | -0,769 | -0,261 |
| Stimuli_vp | Stimuli [intercept] | Vague prior | -0,513 | 0,129 | -0,779 | -0,269 |
| Stimuli_wp | Stimuli [intercept] | Weak prior | -0,514 | 0,13 | -0,777 | -0,268 |
| Measure | Measure [intercept] | Analysis prior | -0,719 | 0,064 | -0,848 | -0,595 |
| Measure_vp | Measure [intercept] | Vague prior | -0,722 | 0,065 | -0,851 | -0,597 |
| Measure_wp | Measure [intercept] | Weak prior | -0,724 | 0,065 | -0,855 | -0,598 |

**Table S6**. Sensitivity analysis of MA 2 (anger)

| **Model** | **Outcome** | **Prior** | **Estimate** | **Std.error** | **Conf.low** | **Conf.high** |
| --- | --- | --- | --- | --- | --- | --- |
| Main | Summary Effect [Global] | Analysis prior | -0,372 | 0,077 | -0,525 | -0,222 |
| Main_vp | Summary Effect [Global] | Vague Prior | -0,373 | 0,076 | -0,524 | -0,226 |
| Main_wp | Summary Effect [Global] | Weak Prior | -0,373 | 0,077 | -0,525 | -0,222 |
| Stimuli | Stimuli [Intercept] | Analysis prior | -0,361 | 0,091 | -0,542 | -0,184 |
| Stimuli_vp | Stimuli [Intercept] | Vague Prior | -0,365 | 0,09 | -0,544 | -0,191 |
| Stimuli_Wp | Stimuli [Intercept] | Weak Prior | -0,365 | 0,092 | -0,549 | -0,188 |
| Measure | Measure [Intercept] | Analysis prior | -0,403 | 0,095 | -0,59 | -0,211 |
| Measure_vp | Measure [Intercept] | Vague Prior | -0,405 | 0,095 | -0,591 | -0,217 |
| Measure_wp | Measure [Intercept] | Weak Prior | -0,406 | 0,094 | -0,59 | -0,224 |

**Table S7**. Sensitivity analysis of MA 2 (disgust)

| **Model** | **Outcome** | **Prior** | **Estimate** | **Std.error** | **Conf.low** | **Conf.high** |
| --- | --- | --- | --- | --- | --- | --- |
| Main | Summary effect [global] | Analysis prior | -0,24 | 0,073 | -0,386 | -0,1 |
| Main_vp | Summary effect [global] | Vague prior | -0,241 | 0,074 | -0,391 | -0,099 |
| Main_wp | Summary effect [global] | Weak prior | -0,24 | 0,072 | -0,386 | -0,099 |
| Stimuli | Stimuli [intercept] | Analysis prior | -0,262 | 0,08 | -0,424 | -0,11 |
| Stimuli_vp | Stimuli [intercept] | Vague prior | -0,263 | 0,081 | -0,429 | -0,108 |
| Stimuli_wp | Stimuli [intercept] | Weak prior | -0,263 | 0,08 | -0,429 | -0,111 |
| Measure | Measure [intercept] | Analysis prior | -0,189 | 0,086 | -0,364 | -0,022 |
| Measure_vp | Measure [intercept] | Vague prior | -0,191 | 0,087 | -0,367 | -0,023 |
| Measure_wp | Measure [intercept] | Weak prior | -0,19 | 0,086 | -0,365 | -0,024 |

**Table S8**. Sensitivity analysis of MA 2 (fear)

| **Model** | **Outcome** | **Prior** | **Estimate** | **Std.error** | **Conf.low** | **Conf.high** |
| --- | --- | --- | --- | --- | --- | --- |
| Main | Summary effect [global] | Analysis prior | -0,37 | 0,079 | -0,538 | -0,223 |
| Main_vp | Summary effect [global] | Vague prior | -0,373 | 0,081 | -0,541 | -0,223 |
| Main_wp | Summary effect [global] | Weak prior | -0,372 | 0,08 | -0,54 | -0,224 |
| Stimuli | Stimuli [intercept] | Analysis prior | -0,375 | 0,095 | -0,573 | -0,195 |
| Stimuli_vp | Stimuli [intercept] | Vague prior | -0,381 | 0,096 | -0,581 | -0,201 |
| Stimuli_wp | Stimuli [intercept] | Weak prior | -0,379 | 0,096 | -0,578 | -0,197 |
| Measure | Measure [intercept] | Analysis prior | -0,377 | 0,087 | -0,556 | -0,212 |
| Measure_vp | Measure [intercept] | Vague prior | -0,38 | 0,088 | -0,558 | -0,21 |
| Measure_wp | Measure [intercept] | Weak prior | -0,381 | 0,088 | -0,56 | -0,211 |

**Table S9**. Sensitivity analysis of MA 2 (happiness/positive)

| **Model** | **Outcome** | **Prior** | **Estimate** | **Std.error** | **Conf.low** | **Conf.high** |
| --- | --- | --- | --- | --- | --- | --- |
| Main | Summary effect [global] | Analysis prior | -0,314 | 0,06 | -0,436 | -0,199 |
| Main_vp | Summary effect [global] | Vague prior | -0,316 | 0,061 | -0,439 | -0,2 |
| Main_wp | Summary effect [global] | Weak prior | -0,317 | 0,061 | -0,44 | -0,198 |
| Stimuli | Stimuli [intercept] | Analysis prior | -0,387 | 0,077 | -0,541 | -0,241 |
| Stimuli_vp | Stimuli [intercept] | Vague prior | -0,39 | 0,078 | -0,544 | -0,238 |
| Stimuli_wp | Stimuli [intercept] | Weak prior | -0,391 | 0,078 | -0,546 | -0,24 |
| Measure | Measure [intercept] | Analysis prior | -0,433 | 0,075 | -0,583 | -0,29 |
| Measure_vp | Measure [intercept] | Vague prior | -0,437 | 0,075 | -0,59 | -0,295 |
| Measure_wp | Measure [intercept] | Weak prior | -0,436 | 0,074 | -0,584 | -0,291 |

**Table S10**. Sensitivity analysis of MA 2 (negative)

| **Model** | **Outcome** | **Prior** | **Estimate** | **Std.error** | **Conf.low** | **Conf.high** |
| --- | --- | --- | --- | --- | --- | --- |
| Main | Summary effect [global] | Analysis prior | -0,203 | 0,085 | -0,38 | -0,044 |
| Main_vp | Summary effect [global] | Vague prior | -0,206 | 0,086 | -0,385 | -0,043 |
| Main_wp | Summary effect [global] | Weak prior | -0,204 | 0,085 | -0,38 | -0,04 |
| Stimuli | Stimuli [intercept] | Analysis prior | -0,171 | 0,115 | -0,401 | 0,058 |
| Stimuli_vp | Stimuli [intercept] | Vague prior | -0,175 | 0,116 | -0,406 | 0,053 |
| Stimuli_wp | Stimuli [intercept] | Weak prior | -0,172 | 0,116 | -0,404 | 0,057 |
| Measure | Measure [intercept] | Analysis prior | -0,421 | 0,131 | -0,683 | -0,171 |
| Measure_vp | Measure [intercept] | Vague prior | -0,429 | 0,132 | -0,696 | -0,175 |
| Measure_wp | Measure [intercept] | Weak prior | -0,429 | 0,133 | -0,697 | -0,173 |

**Table S11**. Sensitivity analysis of MA 2 (neutral)

| **Model** | **Outcome** | **Prior** | **Estimate** | **Std.error** | **Conf.low** | **Conf.high** |
| --- | --- | --- | --- | --- | --- | --- |
| Main | Summary effect [global] | Analysis prior | -0,251 | 0,084 | -0,425 | -0,09 |
| Main_vp | Summary effect [global] | Vague prior | -0,255 | 0,085 | -0,43 | -0,094 |
| Main_wp | Summary effect [global] | Weak prior | -0,254 | 0,085 | -0,429 | -0,09 |
| Stimuli | Stimuli [intercept] | Analysis prior | -0,186 | 0,105 | -0,394 | 0,019 |
| Stimuli_vp | Stimuli [intercept] | Vague prior | -0,188 | 0,106 | -0,393 | 0,02 |
| Stimuli_wp | Stimuli [intercept] | Weak prior | -0,189 | 0,106 | -0,398 | 0,021 |
| Measure | Measure [intercept] | Analysis prior | -0,457 | 0,087 | -0,629 | -0,288 |
| Measure_vp | Measure [intercept] | Vague prior | -0,462 | 0,086 | -0,635 | -0,292 |
| Measure_wp | Measure [intercept] | Weak prior | -0,46 | 0,086 | -0,632 | -0,291 |

**Table S12**. Sensitivity analysis of MA 2 (sadness)

| **Model** | **Outcome** | **Prior** | **Estimate** | **Std.error** | **Conf.low** | **Conf.high** |
| --- | --- | --- | --- | --- | --- | --- |
| Main | Summary effect [global] | Analysis prior | -0,335 | 0,075 | -0,487 | -0,189 |
| Main_vp | Summary effect [global] | Vague prior | -0,336 | 0,074 | -0,486 | -0,191 |
| Main_wp | Summary effect [global] | Weak prior | -0,337 | 0,076 | -0,493 | -0,191 |
| Stimuli | Stimuli [intercept] | Analysis prior | -0,301 | 0,095 | -0,494 | -0,117 |
| Stimuli_vp | Stimuli [intercept] | Vague prior | -0,302 | 0,096 | -0,496 | -0,121 |
| Stimuli_wp | Stimuli [intercept] | Weak prior | -0,304 | 0,095 | -0,5 | -0,123 |
| Measure | Measure [intercept] | Analysis prior | -0,331 | 0,095 | -0,523 | -0,144 |
| Measure_vp | Measure [intercept] | Vague prior | -0,333 | 0,095 | -0,522 | -0,146 |
| Measure_wp | Measure [intercept] | Weak prior | -0,333 | 0,095 | -0,525 | -0,144 |

**Table S13**. Sensitivity analysis of MA 2 (surprise)

| **Model** | **Outcome** | **Prior** | **Estimate** | **Std.error** | **Conf.low** | **Conf.high** |
| --- | --- | --- | --- | --- | --- | --- |
| Main | Summary effect [global] | Analysis prior | -0,263 | 0,08 | -0,426 | -0,106 |
| Main_vp | Summary effect [global] | Vague prior | -0,264 | 0,079 | -0,423 | -0,112 |
| Main_wp | Summary effect [global] | Weak prior | -0,264 | 0,078 | -0,422 | -0,112 |
| Stimuli | Stimuli [intercept] | Analysis prior | -0,325 | 0,094 | -0,512 | -0,141 |
| Stimuli_vp | Stimuli [intercept] | Vague prior | -0,329 | 0,096 | -0,519 | -0,139 |
| Stimuli_wp | Stimuli [intercept] | Weak prior | -0,328 | 0,094 | -0,516 | -0,142 |
| Measure | Measure [intercept] | Analysis prior | -0,247 | 0,094 | -0,431 | -0,062 |
| Measure_vp | Measure [intercept] | Vague prior | -0,249 | 0,095 | -0,437 | -0,06 |
| Measure_wp | Measure [intercept] | Weak prior | -0,248 | 0,095 | -0,436 | -0,06 |
